# A precision therapeutic strategy for hexokinase 1-null, hexokinase 2-positive cancers

**DOI:** 10.1186/s40170-018-0181-8

**Published:** 2018-06-28

**Authors:** Shili Xu, Arthur Catapang, Daniel Braas, Linsey Stiles, Hanna M. Doh, Jason T. Lee, Thomas G. Graeber, Robert Damoiseaux, Orian Shirihai, Harvey R. Herschman

**Affiliations:** 1 Department of Molecular and Medical Pharmacology, David Geffen School of Medicine, University of California, Los Angeles, Los Angeles, CA 90095 USA; 2 Department of Biological Chemistry, David Geffen School of Medicine, University of California, Los Angeles, Los Angeles, CA 90095 USA; 3 UCLA Metabolomics Center, David Geffen School of Medicine, University of California, Los Angeles, Los Angeles, CA 90095 USA; 4 Crump Institute for Molecular Imaging, David Geffen School of Medicine, University of California, Los Angeles, Los Angeles, CA 90095 USA; 5 Jonsson Comprehensive Cancer Center, David Geffen School of Medicine, University of California, Los Angeles, Los Angeles, CA 90095 USA; 6 Division of Endocrinology, Department of Medicine, David Geffen School of Medicine, University of California, Los Angeles, Los Angeles, CA 90095 USA; 7 California NanoSystems Institute, David Geffen School of Medicine, University of California, Los Angeles, Los Angeles, CA 90095 USA; 8 Molecular Biology Institute, David Geffen School of Medicine, University of California, Los Angeles, Los Angeles, CA 90095 USA

**Keywords:** Precision medicine, Warburg effect, Liver cancer, Cancer, Glycolysis, Hexokinase 2, Diphenyleneiodonium, Perhexiline, Fatty acid oxidation, Oxidative phosphorylation, Mitochondria complex-I, Synthetic lethality

## Abstract

**Background:**

Precision medicine therapies require identification of unique molecular cancer characteristics. Hexokinase (HK) activity has been proposed as a therapeutic target; however, different hexokinase isoforms have not been well characterized as alternative targets. While HK2 is highly expressed in the majority of cancers, cancer subtypes with differential HK1 and HK2 expression have not been characterized for their sensitivities to HK2 silencing.

**Methods:**

HK1 and HK2 expression in the Cancer Cell Line Encyclopedia dataset was analyzed. A doxycycline-inducible shRNA silencing system was used to examine the effect of HK2 knockdown in cultured cells and in xenograft models of HK1^−^HK2^+^ and HK1^+^HK2^+^ cancers. Glucose consumption and lactate production rates were measured to monitor HK activity in cell culture, and ^18^F-FDG PET/CT was used to monitor HK activity in xenograft tumors. A high-throughput screen was performed to search for synthetically lethal compounds in combination with HK2 inhibition in HK1^−^HK2^+^ liver cancer cells, and a combination therapy for liver cancers with this phenotype was developed. A metabolomic analysis was performed to examine changes in cellular energy levels and key metabolites in HK1^−^HK2^+^ cells treated with this combination therapy. The CRISPR Cas9 method was used to establish isogenic HK1^+^HK2^+^ and HK1^−^HK2^+^ cell lines to evaluate HK1^−^HK2^+^ cancer cell sensitivity to the combination therapy.

**Results:**

Most tumors express both HK1 and HK2, and subsets of cancers from a wide variety of tissues of origin express only HK2. Unlike HK1^+^HK2^+^ cancers, HK1^−^HK2^+^ cancers are sensitive to HK2 silencing-induced cytostasis. Synthetic lethality was achieved in HK1^−^HK2^+^ liver cancer cells, by the combination of DPI, a mitochondrial complex I inhibitor, and HK2 inhibition, in HK1^−^HK2^+^ liver cancer cells. Perhexiline, a fatty acid oxidation inhibitor, further sensitizes HK1^−^HK2^+^ liver cancer cells to the complex I/HK2-targeted therapeutic combination. Although HK1^+^HK2^+^ lung cancer H460 cells are resistant to this therapeutic combination, isogenic HK1^KO^HK2^+^ cells are sensitive to this therapy.

**Conclusions:**

The HK1^−^HK2^+^ cancer subsets exist among a wide variety of cancer types. Selective inhibition of the HK1^−^HK2^+^ cancer cell-specific energy production pathways (HK2-driven glycolysis, oxidative phosphorylation and fatty acid oxidation), due to the unique presence of only the HK2 isoform, appears promising to treat HK1^−^HK2^+^ cancers. This therapeutic strategy will likely be tolerated by most normal tissues, where only HK1 is expressed.

**Electronic supplementary material:**

The online version of this article (10.1186/s40170-018-0181-8) contains supplementary material, which is available to authorized users.

## Background

Precision medicine depends on the identification of a unique molecular cancer subtype that may exist across tumors with different origins. The recent Food and Drug Administration (FDA) approval of Keytruda for treatment of a wide range of advanced cancers with the common biomarkers microsatellite instability-high (MSI-H) or mismatch repair deficient (dMMR) illustrates this concept of phenotype/genotype commonality, rather than tissue of origin, for a common therapeutic approach to subclasses of cancers from tissues of varying origin [1]. Larotrectinib, which targets the rare tropomyosin receptor kinase (TRK) fusion mutation, is another example of precision cancer medicine; larotrectinib has demonstrated efficacy in cancers from different tissues that share TRK fusion mutations [2]. FDA approval is currently being sought for the larotrectinib treatment of adult and pediatric TRK fusion cancers, based on molecular makeup rather than the tissue of origin [3].

Most cancers increase glycolysis (the “Warburg effect”), a metabolic event postulated to ensure sufficient supplies of energy (ATP), reducing equivalents (NADPH), and/or biochemical building blocks for cell growth and proliferation [4]. However, because of the conserved nature of the glycolytic pathway in normal tissues, global systemic inhibition of glycolysis results in adverse effects that make this approach of limited value; selective inhibition of cancer-driven glycolysis will be required for clinical cancer therapy.

The hexokinase (HK) enzymes, encoded by four genes (HK1/2/3/4), catalyze glucose phosphorylation, the first enzymatic step in glycolysis [5]. Most adult tissues use HK1 for glycolysis. HK3 is inhibited by physiological concentrations of glucose. HK4, also known as glucokinase (GCK), is expressed in hepatocytes, pancreatic β-cells, and glucose-sensing neurons. HK2 is expressed primarily in embryonic tissues and in adult muscle and adipose tissues. In addition, HK2 is expressed in a wide range of cancers [6–9], from tissues that normally express only HK1. HK2 gene deletion in adult mice does not significantly affect normal tissues [6]. Consequently, selective HK2 inhibitors have recently been developed [10], under the assumption that targeted HK2 inhibition will reduce progression of HK2-positive cancers and have minimal adverse effects. However, most previous studies on HK2 in cancer did not examine the contribution of HK1 to cancer glycolysis [6–9, 11]. Cancer subtypes with differential HK1/HK2 expression have not been characterized.

In addition to enhanced glycolysis, other modes of energy generation are utilized to support biological processes in cancer cells; these alternative energy-generating sources include oxidative phosphorylation and fatty acid oxidation [12, 13]. The availability and use of multi-source energy generation alternatives suggests both the flexibility of cancer cells in reprogramming their required energy generation under metabolic stress and their abilities to escape from alternative modes of energy generation blockade using monotherapies. Combination therapies that target compensatory energy metabolism pathways in cancer cells, but are tolerated by normal tissues, have not yet achieved clinical success, due to the conserved nature of energy metabolism shared by most cancer and normal cells.

We have developed a combination therapy specifically effective in cancers with the HK1^−^HK2^+^ molecular signature, a subset of cancers which originate from a wide variety of tissues. Using an HK1^−^HK2^+^ subpopulation of liver cancers as an example, we developed a synergistic combination of HK2 inhibition and partial inhibition of mitochondrial complex I and fatty acid oxidation to achieve synthetic lethality of these HK1^−^HK2^+^ tumors. However, like HK1^+^ cells/tissues, HK1^+^HK2^+^ cancers are not susceptible to this therapy, despite the reduction in their HK2-driven glycolytic activity. Our findings warrant optimization of the triple-combination therapy as a precision medicine for further clinical development to treat HK1^−^HK2^+^ cancers, regardless of their tissues of origin.

## Methods

### Cell lines and tissue samples

Hep3B, HepG2, H460, H1299, HT29, HCT116, SW620, and Caco2 cells were from the American Type Culture Collection (ATCC). Huh7, JHH7, JHH5, and MDAMB468 cells were provided by Drs. Dennis Slamon and Richard Finn (UCLA). SUM159, MDAMB231, and HCC1937 cells were provided by Dr. Heather Christofk (UCLA). A549, SKOV3, OVCAR5, and HEY cells were provided by Dr. Caius Radu (UCLA). Hs766T, MiaPaca2, and Panc1 were provided by Dr. Timothy Donahue (UCLA). LNCaP, DU145, and PC3 cells were provided by Dr. Hong Wu (UCLA). 786-0 cells were provided by Dr. Allan Pantuck (UCLA). All cells used for the experiments were between passages 3 and 20. Cell lines were routinely authenticated based on morphology, growth characteristics, and HK expression profiles according to the CCLE gene expression dataset. All cells were maintained in RPMI1640 + 10% FBS, at 37 °C in 5% CO_2_/95% air. Cells were routinely checked for *Mycoplasma* contamination by using MycoAlert (Lonza). Frozen human liver and liver cancer samples were provided by the UCLA Translational Pathology Core Laboratory.

### High-throughput screen (HTS) for compounds synergistic with HK2 knockdown in cell growth inhibition

In the primary HTS screening, libraries of 3205 drug-like small molecules and 119 FDA-approved oncology drugs were screened for their ability to inhibit the growth of Hep3B/shHK2^DOX^ cells in the presence of DOX. Hep3B/shHK2^DOX^ cells were pretreated with DOX for 48 h, then seeded in 384-well plates with 700 cells per well, and treated with DOX and individual library members at 10 μM for 72 h. Relative numbers of viable cells in response to different treatments were determined by the CellTiter-Glo assay (Promega). Compounds with *z* score < − 3 were selected for subsequent secondary screening. In the secondary screening, Hep3B/shHK2^DOX^ cells with or without 48-h DOX pretreatment, were treated subsequently with the selected compounds in dose response curves (DRCs 10, 2.5, 0.625, 0.156, 0.039, 0.010, 0.0024, and 0.0006 μM) for 72 h. Relative numbers of viable cells were determined by the alamarBlue assay (Invitrogen).

### Media metabolite measurement

Medium was collected from culture plates and analyzed for glucose, lactate, and glutamine concentrations using a Biomedical Bioprofile Analyzer (Nova Biomedical). Cells seeded in 6-well plates received treatments described in the “Results” section and the figure legends. Twenty-four hours before the analysis, the media were refreshed. Medium added to wells with no cells was used as a blank control. After 24-h incubation, 1 ml of medium was collected from each sample and the blank control, and media samples were analyzed in the Bioprofile Analyzer. Values were normalized to cell number and time intervals. DPI was purchased from Cayman Chemical (#81050). PER was purchased from Cayman Chemical (#16982). FDG was purchased from Omicron Biochemicals Inc. (#GLC-010).

### In vivo assessment of treatment efficacy and safety

Nu/nu mice (Jackson Laboratory) were used for in vivo efficacy and safety studies. Mice were fed ad libitum and kept in air-conditioned rooms at 20 ± 2 °C with a 12-h light-dark period. Animal care and manipulation were in accordance with the Guidelines for the Care and Use of Laboratory Animals. Cells (Hep3B, 5 × 10^6^; Huh7, 2 × 10^6^; HepG2, 2 × 10^6^; H460, 1 × 10^6^) in 100 μl RPMI/matrigel (1:1) were implanted subcutaneously in nu/nu mice under aseptic conditions. Body weight was measured biweekly. The body condition score (BCS) was also used to evaluate animal health. Tumor growth was assessed by biweekly measurement of tumor diameters with a vernier caliper. Tumor volume (mm^3^) = *D* × *d*^2^/2, where *D* and *d* are the longest and shortest diameters, respectively. After subcutaneous tumor cell inoculation, tumors were allowed to grow to a volume of 200 mm^3^. We chose tumors of this size because, in our experience, tumor xenografts of smaller sizes may regress. Mice were then randomly assigned into groups as indicated in the “Results” section and the figure legends. DOX was given in the diet (625 mg DOX per kg diet, daily DOX 1.6–2.7 mg in 3–5 g diet per mouse). DPI (#D491550, Toronto Research Chemicals) and PER were dissolved in DMSO as stock solutions at 20 and 300 mg/ml, respectively, diluted in 5% (*w*/*v*) hydroxyl-propyl-beta-cyclodextrin (Sigma) as injection solutions, and given daily by intraperitoneal (i.p.) injection at 2 and 30 mg/kg, respectively.

### Statistics

Student’s t-test was used for statistical analysis. *P* values were determined by Prism 5 (GraphPad Software, Inc.). Differences were considered statistically significant at *P* < 0.05.

### Study approval

All reported animal studies were approved by the UCLA Chancellor’s Animal Research Committee (ARC).

## Results

### The conversion of liver to liver cancer is accompanied by extinction of HK4 expression and tumor expression of either HK2 alone or HK1 and HK2

**Fig. 1:**
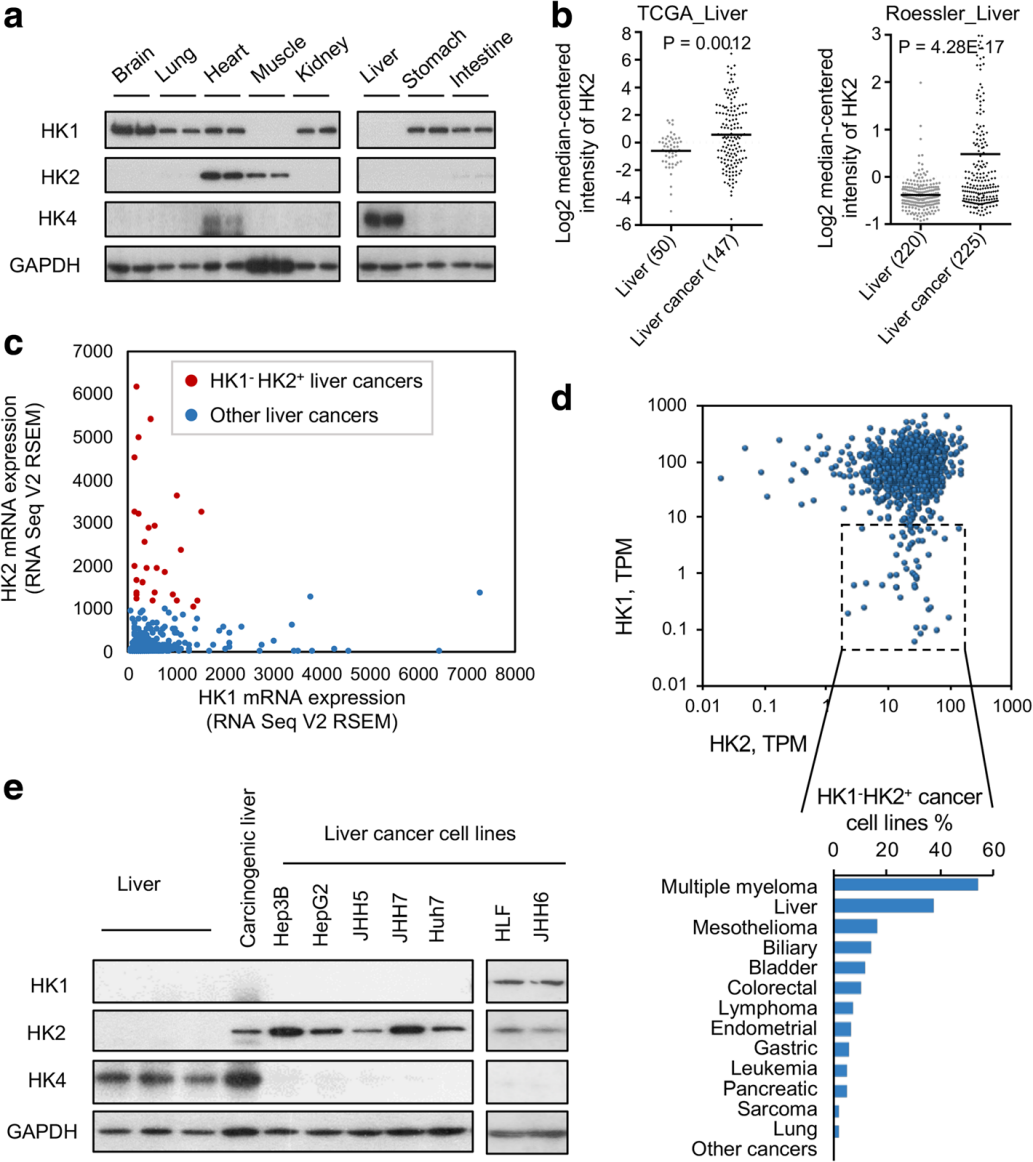
Subsets of cancers from a variety of tissues of origin are HK1^−^HK2^+^. **a** HK1, HK2, and HK4 protein levels in organs. Organs were collected from two C57BL/6 male mice. **b** HK2 expression is upregulated in clinical liver cancer samples relative to normal liver. The TCGA_Liver dataset was obtained from OASIS (http://oasis-genomics.org), and the Roessler_Liver dataset was obtained from Oncomine (http://www.oncimine.com). Numbers after sample names indicate sample size. Bar: mean value. **c** HK1 and HK2 mRNA expression profiles in the TCGA_Liver Cancer dataset. Data were obtained from the cBioPortal (http://www.cbioportal.org). RSEM RNA-Seq by Expectation Maximization. **d** Identification of HK1^−^HK2^+^ cancer cell lines present in the Cancer Cell Line Encyclopedia (CCLE) collection (http://www.cbioportal.org). Upper panel, HK1 and HK2 mRNA expression of 935 cell lines with RNASeq data in the CCLE dataset. Each data point represents one cell line. The box indicates “HK1^−^HK2^+^” cell lines, defined as HK2 > 1 TPM and HK1 < 10 TPM. TPM transcripts per million. Lower panel, distribution of the CCLE HK1^−^HK2^+^ cancer cell lines in a broad spectrum of cancers of different tissues of origin. **e** Hexokinase isoform expression in four human samples and seven human liver cancer cell lines

Our initial goal was to identify a tumor population likely to be highly sensitive to HK2 inhibition or silencing, to optimize evaluation of targeting HK2 as a therapeutic modality. Unlike most adult tissues, which express HK1 to drive glycolysis, the liver expresses HK4 (also known as glucokinase) and does not express either HK1 or HK2 (Fig. 1a). Liver cancers, however, no longer express HK4; instead, analysis of datasets of liver cancer samples [14, 15] revealed that, like many other cancers, HK2 is expressed in many liver tumors (Fig. 1b). Moreover, analysis of HK1 and HK2 expression in the TCGA liver cancer dataset demonstrates that a substantial portion (83%) of HK2^+^ (HK2 RSEM > 1000) liver cancers are deficient in HK1 expression (HK1 RSEM < 1000) (Fig. 1c).

Because tumor biopsies are likely to have normal tissue contamination, it is not possible to evaluate tumors in the TCGA collection from other tissues of origins for tumor HK1 and HK2 expression; the presence of HK1 derived from most normal tissues would distort the estimate of HK1 tumor-derived levels in tumor biopsies. In contrast, contamination with normal liver, which does not express either HK1 or HK2, would not affect the evaluation of HK1 and HK2 expression in liver tumor samples.

### Tumors of multiple origin contain HK1^−^HK2^+^ and HK1^+^HK2^+^ subsets

To confirm and extend the observations regarding the presence of HK1^−^HK2^+^ liver cancers and to determine whether HK1^−^HK2^+^ subsets exist in cancers from other tissues of origin, we examined the frequencies of HK1^−^HK2^+^ and HK1^+^HK2^+^ subsets in established cancer cell lines of the Cancer Cell Line Encyclopedia (CCLE) collection [16]. Subsets of cancer cell lines exhibiting the HK1^−^HK2^+^ molecular characteristic are present, at varying frequencies, across a broad spectrum of cancer types from a wide variety of tissues of origin (Fig. 1d).

We chose to use liver cancer cell lines to investigate HK2 as a therapeutic target because of (i) the substantial contributions of both HK1^−^HK2^+^ and HK1^+^HK2^+^ tumor cell lines to the liver CCLE population, (ii) the lack of effective treatment for liver cancers, and (iii) the prediction of liver cancer as the third most lethal cancer in the USA in the next decade [17]. To confirm and extend these observations, we evaluated the expression of HK1 and HK2 in human liver samples and in a collection of liver cancer cell lines (Fig. 1e). At the protein level, all established human liver cancer cell lines we examined express HK2 and lack HK4. Moreover, as expected from the TCGA data, these liver cancer cell lines fall into two subpopulations: HK1^−^HK2^+^ (Hep3B, HepG2, JHH5, JHH7, Huh7) and HK1^+^HK2^+^ (HLF, JHH6) (Fig. 1e). We used these liver cancer cell lines to examine the therapeutic efficacy of the targeted inhibition of HK2.

### HK2 shRNA-silencing mediated inhibition of proliferation and colony formation in HK1^−^HK2^+^ cancer cells, but not HK1^+^HK2^+^ cancer cells, is a property of cancer cells from a variety of tissues of origin

**Fig. 2:**
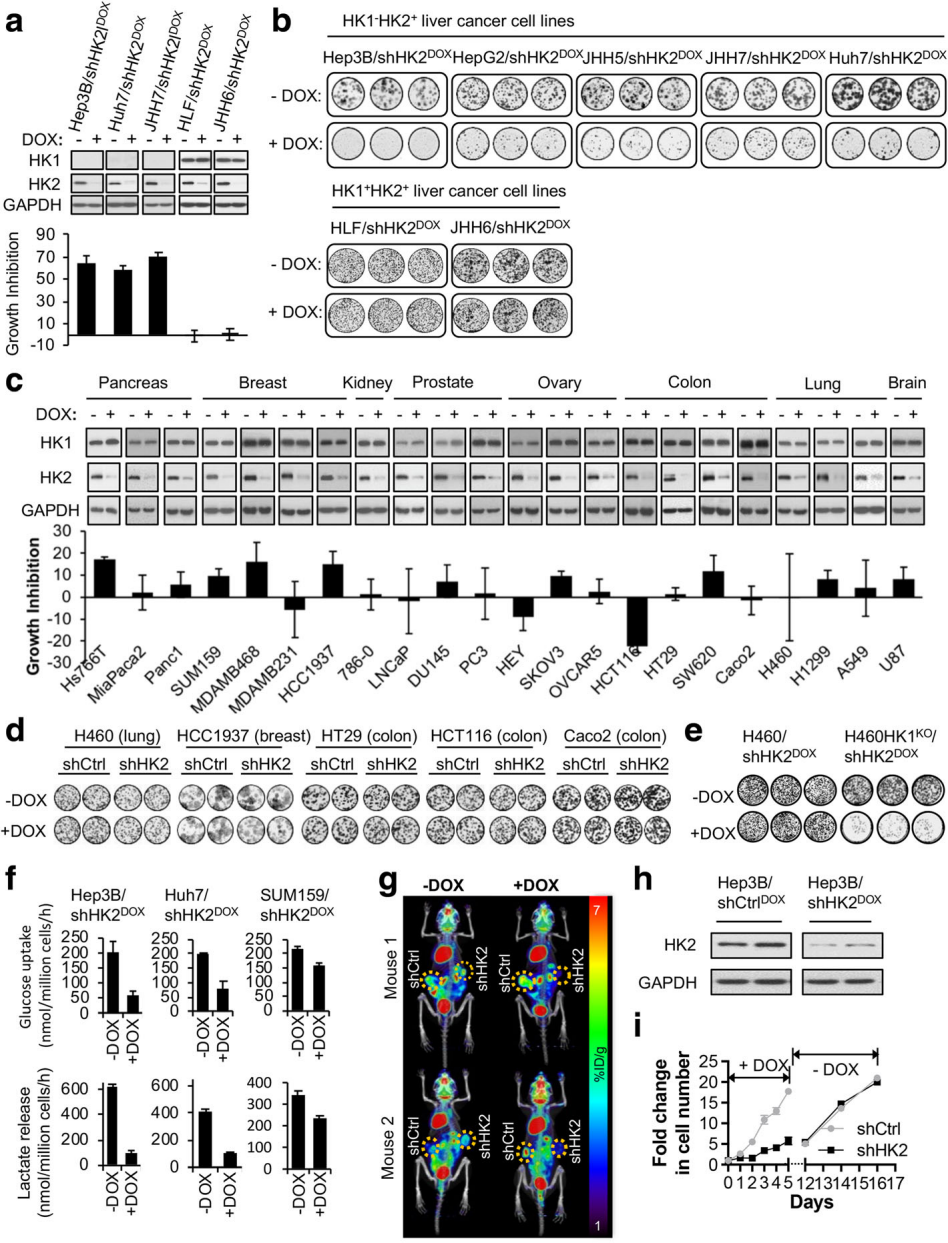
HK1^−^HK2^+^ liver cancer cells are sensitive to HK2 knockdown-induced growth inhibition. **a** HK1^−^HK2^+^ liver cancer cells are sensitive to HK2 knockdown-induced growth inhibition. Top panel, HK2 knockdown by DOX-inducible shHK2^DOX^ after 72 h DOX (25 ng/ml) induction. Bottom panel, after a 7-day exposure to DOX to induce shRNA expression, relative cell numbers were measured using the MTT assay. Data are expressed as “growth inhibition” [100 × (1 − MTT assay values of DOX-treated cells/MTT assay values of untreated cells)]. **b** Effect of DOX-induced HK2 knockdown on colony formation by HK1^−^HK2^+^ and HK1^+^HK2^+^ liver cancer cells. Triplicate wells of each cell line are shown, stained at 15–20 days after plating. **c**, **d** DOX-induced HK2 silencing has limited or no effect on **c** cell proliferation or **d** colony formation in HK1^+^HK2^+^ cancer cells, regardless of tissue of origin. **e** DOX-induced shRNA HK2 knockdown inhibited colony formation by H460HK1^KO^/shHK2^DOX^ isogenic cells but not by isogenic H460/shHK2^DOX^ cells. **f** HK2 knockdown inhibits glucose consumption (upper panels) and lactate production (lower panels) in Hep3B/shHK2^DOX^, Huh7/shHK2^DOX^, and SUM159/shHK2^DOX^ cells. Triplicate wells of cells were exposed to DOX or vehicle for 72 h*. Media* were then refreshed and, after 24 h, glucose consumption and lactate production were measured. **g** HK2 knockdown decreases ^18^F-FDG/PET signal in a mouse subcutaneous Hep3B/shHK2^DOX^ xenograft model. Tumors on the left flank: Hep3B/shCtrl^DOX^. Tumors on the right flank: Hep3B/shHK2^DOX^. ^18^F-FDG PET/CT scans were performed for each mouse before and after mice were switched to a DOX diet for 4 days. **h** Following the PET scans in panel **g**, HK2 knockdown was confirmed in tumor extracts by Western blotting. **i** Cytostasis induced in HK1^−^HK2^+^ liver cancer cells by HK2 knockdown is reversible. Hep3B/shHK2^DOX^ cells and Hep3B/shCtrl^DOX^ cells were treated with DOX for 2 days then re-plated in equal numbers in 96-well plates on day 0 in the continued presence of DOX. Cell proliferation during days 0–5 in the presence of DOX was measured (left panel). Cells from the two conditions (+DOX, −DOX) were pooled and cultured in DOX-free medium for 7 days (days 5–12) to allow the degradation of shRNAs in the cells and re-expression of HK2. The cells were then re-plated in 96-well plates and grown in the absence of DOX to generate the growth curves for days 12–16 (right panel). Relative cell proliferation was measured by the MTT assay and presented as fold change compared to the value on day 0. Each data point in panels **a**, **c**, and **f** represents mean ± SD of triplicate samples

To examine tumor cell growth inhibition in response to HK2 knockdown, we investigated the consequences of expressing an HK2-targeted doxycycline (DOX)-inducible shRNA in our panel of HK1^−^HK2^+^ and HK1^+^HK2^+^ liver cancer cell lines. An inducible HK2 shRNA was used to avoid the potential selection for cells resistant to HK2 silencing. shHK2 expression suppressed both proliferation (Fig. 2a, lower panel) and colony formation (Fig. 2b) of the HK1^−^HK2^+^ liver cancer cell lines. However, in contrast to the results obtained with the HK1^−^HK2^+^ liver cancer cell lines, shHK2 expression in the HK1^+^HK2^+^ liver cancer cell lines had no significant effect on either cell proliferation (Fig. 2a, lower panel) or colony formation (Fig. 2b). These results were validated using two additional shRNA sequences for HK2 (Additional file 1: Figure S1A and S1B).

The lack of an shHK2 inhibitory response for HK1^+^HK2^+^ cancer cells is not unique to liver cancer cells; shHK2 inhibition of HK2 expression is unable to prevent proliferation or colony formation in HK1^+^HK2^+^ tumor cell lines derived from lung, breast, or colon cancers (Fig. 2c, d) as well as HK1^+^HK2^+^ liver cancer cells. In addition, we used CRISPR Cas9 HK1 deletion to create isogenic HK1^−^HK2^+^ and HK1^+^HK2^+^ H460 lung cancer cell lines (Additional file 1: Figure S1C). DOX-induced shHK2 expression in the HK1^−^HK2^+^ H460 cells suppressed colony formation but had no effect on isogenic HK1^+^HK2^+^ H460 cell colony formation (Fig. 2e); conversion of an HK1^+^HK2^+^ cancer cell to an isogenic HK1^−^HK2^+^ cancer cell has no effect on cell growth or proliferation but converts cells resistant to shHK2-mediated inhibition of proliferation or colony formation to cells sensitive to shHK2 inhibition.

Both HK1 and HK2 drive cancer aerobic glycolysis—glucose consumption and lactic acid production. Consequently, HK2 knockdown by DOX-induced shHK2 resulted in a greater reduction of both glucose consumption and lactate production more pronounced in HK1^−^HK2^+^ cancer cells (Hep3B, Huh7) compared to HK1^+^HK2^+^ cancer cells (SUM159) (Fig. 2f). However, before proceeding further with consideration of HK2-targeted therapy, we wanted to determine if reduction of HK2 activity in HK1^−^HK2^+^ cells would also reduce glucose consumption in vivo. In mouse subcutaneous Hep3B/shHK2^DOX^ tumor xenografts, HK2 knockdown by a 4-day DOX treatment in the diet reduced tumor glucose consumption as determined non-invasively by ^18^F-fluorodeoxyglucose (FDG)/positron emission tomography (PET) imaging [18, 19] (Fig. 2g, h).

While HK2 knockdown in HK1^−^HK2^+^ liver cancer cells substantially reduced cell proliferation, the majority of the cells remained alive (Additional file 1: Figure S1D); after removing DOX from cultured Hep3B/shHK2^DOX^ cells, the DOX-treated HK1^−^HK2^+^ cells were able to recover from HK2-inhibited cytostasis and resumed proliferation (Fig. 2i). These data suggest that monotherapy with selective HK2 inhibitors in HK1^−^HK2^+^ tumors is unlikely to be curative, either in preclinical models or in clinical applications; identification of synergistic or synthetically lethal agents in combination with HK2 inhibition will be necessary for HK2-targeted therapy of HK1^−^HK2^+^ tumors.

### Diphenyleneiodonium (DPI), in combination with HK2 knockdown, is synthetically lethal for HK1^−^HK2^+^ liver cancer cells

**Fig. 3:**
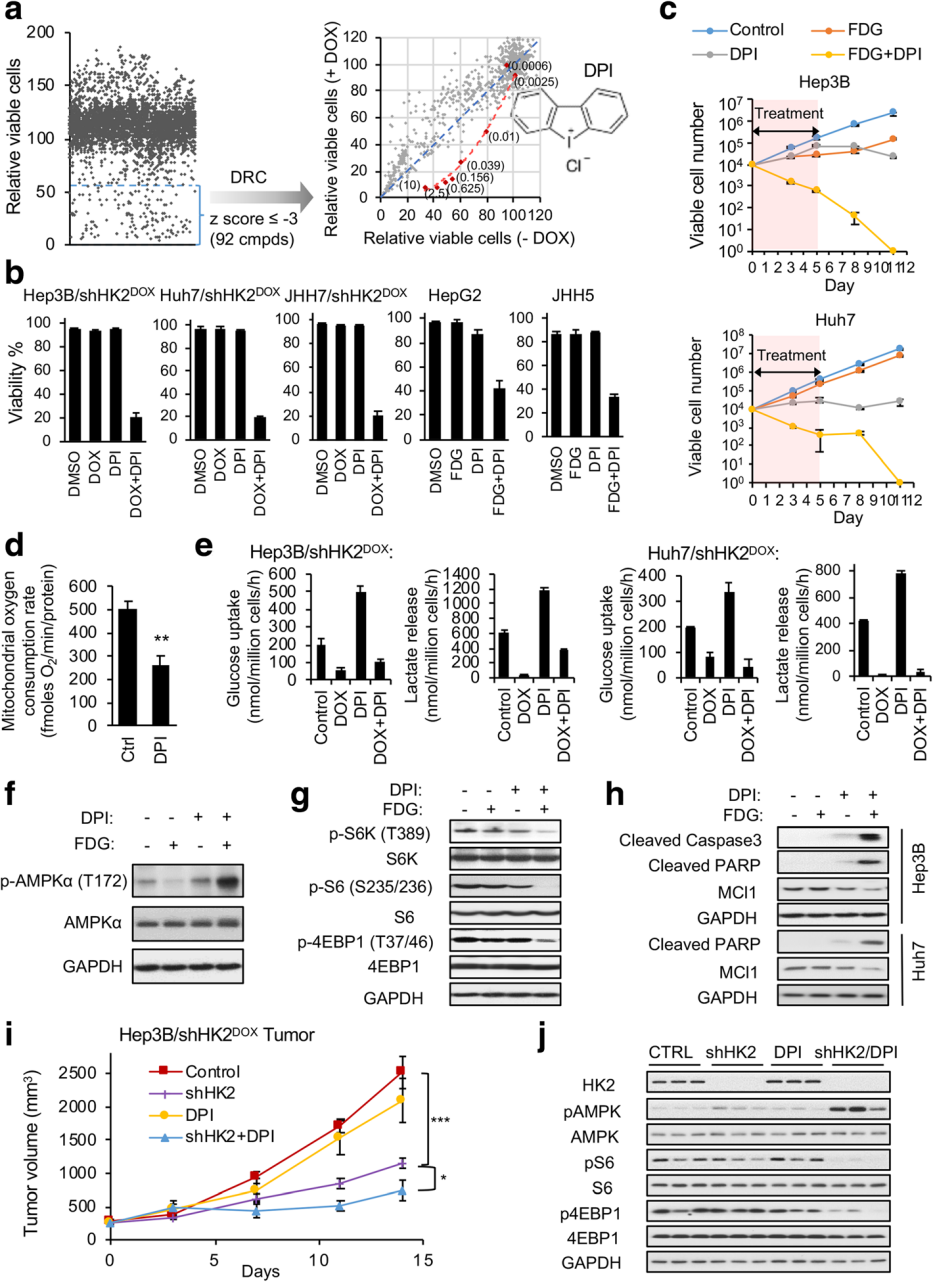
DPI is a synthetically lethal partner in combination with HK2 knockdown or inhibition in HK1^−^HK2^+^ liver cancer cells. **a** A high-throughput screen of 3205 drug-like compounds and 119 FDA-approved oncology drugs identified DPI as the best synergistic agent with HK2 knockdown in Hep3B/shHK2^DOX^ cells. Primary screening of all compounds at 10 μM was performed in combination with DOX-induced HK2 knockdown in Hep3B/shHK2^DOX^ cells. Growth inhibition was measured by the CellTiter-Glo assay (left panel). Ninety-two compounds with *z* scores ≤ − 3 were tested in dose response curves (dose response concentrations 0, 0.0006, 0.0025, 0.01, 0.039, 0.156, 0.625, and 2.5 μM) in Hep3B/shHK2^DOX^ cells cultured with or without DOX. Viable cells were normalized to DMSO-treated cells with or without DOX, respectively (right panel). The dots represent compounds at the tested concentrations. The red line connects the individual DPI concentration points. The chemical structure of DPI is shown. **b** DPI is synthetically lethal with HK2 knockdown or inhibition in HK1^−^HK2^+^ liver cancer cells. DOX/DPI 2-day DOX pretreatment prior to 72 h DPI (100 nM) treatment. FDG/DPI 72 h FDG (1 mM) and/or DPI (100 nM) treatments. Viability percentages were determined by trypan blue staining. **c** FDG/DPI combination irreversibly inhibits Hep3b and Huh7 HK1^−^HK2^+^ liver cancer cell proliferation. Viable cells were determined by trypan blue staining. **d** DPI decreases the oxygen consumption rate (OCR) of Hep3B cells. Cells were exposed to 100 nM DPI or vehicle for 2 h before OCR measurements. **e** DPI increases glycolysis of HK1^−^HK2^+^ liver cancer cells. Cells were pretreated with vehicle or DOX for 72 h prior to exposure to DPI (100 nM). Twenty-four hours later, culture media were analyzed for glucose and lactate. **f**, **g** FDG/DPI treatment activates AMPKα (**f**) and inactivates the mTOR pathway (**g**) in Hep3B cells. Cells were treated with 1 mM FDG and/or 100 nM DPI for 4 h. **h** FDG/DPI treatment triggers apoptosis. Hep3B HK1^−^HK2^+^ cells were treated with FDG (1 mM) and/or DPI (100 nM) for 24 h prior to apoptosis analyses. **i** DPI (2 mg/kg) enhances inhibition of tumor progression in response to HK2 silencing in Hep3B/shHK2^DOX^ tumors. Mice-bearing tumor xenografts were treated when tumors reached 200 mm^3^ (day 0). **j** Effects of HK2 knockdown and/or DPI (2 mg/kg) treatment on HK2 expression, AMPKα activation, and mTOR pathway inactivation in the xenograft Hep3B/shHK2^DOX^ tumors described in panel **i**. Each data point in **a**–**c** and **e** represents mean ± SD of triplicate samples, and each data point in **d** and **i** represents mean ± SEM of five samples. *P < 0.05, ***P < 0.001

To search for partners synthetically lethal with HK2 silencing in HK1^−^HK2^+^ cancer cells, 119 FDA-approved oncology drugs and 3205 drug-like compounds were screened, with or without DOX-induced shHK2 HK2 knockdown, in Hep3B/shHK2^dox^ cells (Fig. 3a, Additional file 2: Table S1). None of the 119 FDA-approved oncology drugs were synergistic with HK2 knockdown. DPI was the molecule best able, in combination with HK2 knockdown, to reduce the number of viable Hep3B/shHK2^dox^ cells (Fig. 3a and Additional file 1: Figure S2A). Because HK2 is the only HK isoform expressed in Hep3B cells, FDG (an inhibitor of both HK1 and HK2) can be used as an “HK2-targeted inhibitor,” in place of HK2 shRNA knockdown, to demonstrate the synergistic effect between HK2 inhibition and DPI for HK1^−^HK2^+^ Hep3B growth inhibition (Additional file 1: Figure S2B). Using the Chou-Talalay method [20], we confirmed that the combination of DPI and FDG resulted in substantial synergy in all five HK1^−^HK2^+^ liver cancer cell lines tested (Additional file 2: Table S2). While shHK2, FDG, or DPI as single agents were not cytotoxic at the concentrations used in this experiment, they slow cell proliferation; in contrast, the combination of either DPI/shHK2 or DPI/FDG caused synthetic lethality in these HK1^−^HK2^+^ liver cancer cells (Fig. 3b, c and Additional file 1: Figure S2C). However, the synergism/synthetic lethality between HK2 knockdown and DPI is restricted to HK1^−^HK2^+^ cancer cells and is not observed in HK1^+^HK2^+^ liver cancer cells or in HK1^+^HK2^+^ cancer cells from other tissues (Additional file 1: Figure S2D). It is important to note that we chose to use FDG instead of 2-deoxy-D-glucose (2DG, a commonly used reagent to study glycolysis) because 2DG inhibits both HK1/2-driven glycolysis and N-linked glycosylation, whereas FDG only inhibits HK1/2-driven glycolysis [21]. As a result, 2DG is toxic as a single agent but FDG is not [21].

DPI has been reported to inhibit NADPH oxidase (NOX) [22], nitric oxide synthase (NOS) [23], and mitochondrial complex I [24]. Tested in combination with HK2 knockdown, neither NOX inhibitors (GKT137831, apocynin; Additional file 1: Figure S3A) nor NOS inhibitors (L-NAME or L-NNA) (Additional file 1: Figure S3B) show synergy in HK1^−^HK2^+^ liver cancer cells. In contrast, the complex I inhibitor rotenone (ROT) is synthetically lethal in combination with HK2 knockdown (Additional file 1: Figure S3C). In addition, DPI treatment reduced, but did not eliminate, mitochondrial oxygen consumption (Fig. 3d), consistent with its inhibition of mitochondrial complex I, and increased cellular demand for glycolysis (Fig. 3e). These results indicate that DPI synergizes with HK2 silencing/inhibition in HK1^−^HK2^+^ liver cancer cells through the inhibition of mitochondrial complex I, reducing ATP production from the electron transport chain (ETC). The synergistic reduction in cellular energy levels by FDG/DPI or FDG/ROT triggered the activation of the cellular energy sensor AMPKα (Fig. 3f and Additional file 1: Figure S3D), suppressed the energy-dependent mTOR pathway (Fig. 3g and Additional file 1: Figure S3E) [25], and induced apoptosis (Fig. 3h and Additional file 1: Figure S3F).

In a mouse xenograft model of HK1^−^HK2^+^ Hep3B/shHK2^DOX^ tumor progression, DOX-induced HK2 knockdown initiated when the tumors reached 200 mm^3^ significantly reduced tumor growth (Fig. 3i). Although DPI alone had no significant effect on tumor growth, the DPI/shHK2 combination was significantly more effective in reducing tumor growth when compared to the effect of either shHK2 or DPI alone (Fig. 3i). The Hep3B/shHK2^DOX^ tumors, in which HK2 was reduced to undetectable levels, did not express other active HK isoforms to compensate for glycolysis (Additional file 1: Figure S4). The combination shHK2/DPI treatment resulted in increased levels of phosphorylated AMPKα and dephosphorylation of S6 and 4EBP1 in xenograft Hep3B/shHK2^DOX^ tumors (Fig. 3j), demonstrating the reduction in tumor energy production induced by the shHK2/DPI combination therapy.

### Inhibition of fatty acid oxidation sensitizes HK1^−^HK2^+^ liver cancer cells to the FDG/DPI combination

**Fig. 4:**
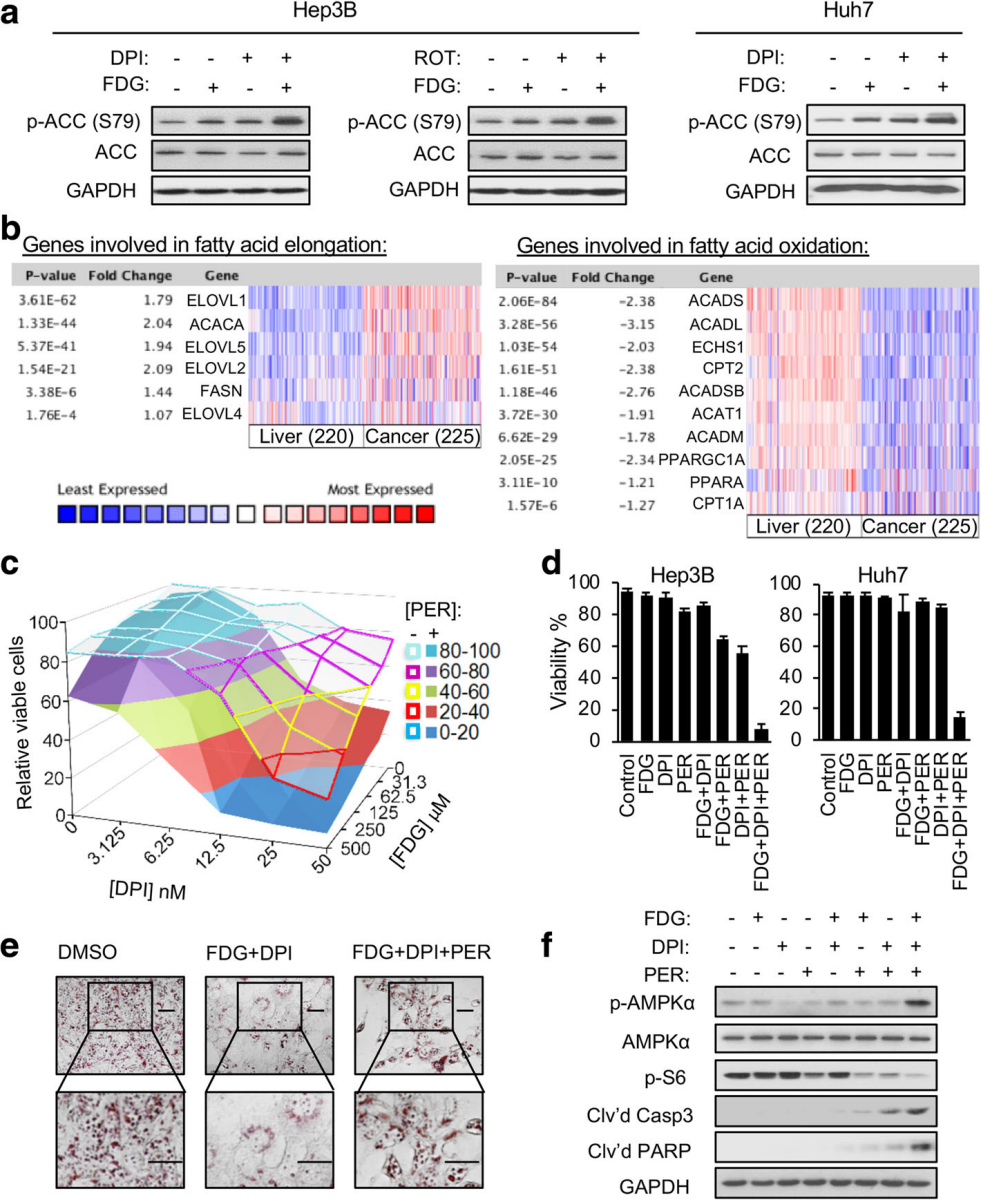
Inhibition of fatty acid oxidation sensitizes HK1^−^HK2^+^ liver cancer cells to the cytotoxicity of the HK2 inhibition/DPI combination. **a** FDG/DPI or FDG/ROT combinations induce ACC phosphorylation. HK1^−^HK2^+^ liver cancer cells were treated with the indicated drug(s) for 24 h prior to analysis of ACC phosphorylation. FDG, 1 mM. DPI, 100 nM. ROT, 100 nM. **b** Clinical liver cancer samples show upregulated expression of genes involved in fatty acid elongation and downregulated expression of genes involved in FAO compared to normal liver tissues. Data were obtained from Oncomine (http://www.oncomine.com). **c** PER sensitizes Hep3B cells to FDG/DPI treatment. Cells were treated with FDG, plus or minus 5 μM PER, for 72 h and then assayed for viable cells by the MTT assay. Open surface, [DPI + FDG + vehicle] treatment. Filled surface, [DPI + FDG + PER] treatment. **d** PER sensitizes HK1^−^HK2^+^ liver cancer cells to the cytotoxicity of FDG/DPI treatment. Triplicate wells of (left) Hep3B or (right) Huh7 cells were treated with FDG (250 μM), DPI (15 nM), and/or PER (5 μM) for 72 h, followed by trypan blue staining to determine cell viability. Viability % = 100% × (trypan blue negative cells/total cells). Data are means ± SD. **e** PER rescues FDG/DPI-induced lipid droplet depletion in Hep3B cells. Hep3B cells were treated with DMSO, FDG (1 mM)/DPI (100 nM), or FDG (1 mM)/DPI (100 nM)/PER (5 μM) for 24 h, followed by Oil Red staining of intracellular lipid droplets. Representative images with indicated treatments are shown. Scale bars, 30 μM. **f** FDG, DPI, and/or PER modulation of AMPKα activation, mTOR pathway inactivation, and apoptosis induction in Hep3B/shHK2^DOX^ cells. Cells were treated with FDG (250 μM), DPI (15 nM), and/or PER (5 μM) for 24 h, and cell extracts were examined by Western blotting

Acetyl-CoA carboxylase (ACC), the first and rate-limiting enzyme in fatty acid biosynthesis, regulates fatty acid oxidation (FAO) [26]. Both the FDG/DPI and FDG/ROT combinations triggered ACC phosphorylation in Hep3B and Huh7 HK1^−^HK2^+^ liver cancer cells (Fig. 4a and Additional file 1: Figure S5A), an indicator of ACC inhibition. These data suggest the FDG/DPI combination treatment shifts the balance of cellular metabolism in HK1^−^HK2^+^ cancer cells from fatty acid elongation, which is used for membrane synthesis and cell proliferation, to FAO to provide additional fuel for energy generation through residual mitochondrial respiration (Fig. 3d) as a survival strategy. When compared to normal liver, liver cancers upregulate expression of genes involved in fatty acid elongation and downregulate expression of genes involved in FAO (Fig. 4b). These data also suggest FAO inhibitors might sensitize HK1^−^HK2^+^ cancer cells to the combination of DPI and HK2 inhibition but could be tolerated by HK4-expressing hepatocytes and by other normal tissues which express HK1.

Perhexiline (PER), a FAO inhibitor in clinical use as an anti-angina drug in Australia and Asia and currently in clinical trials in the USA [13], sensitized HK1^−^HK2^+^ Hep3B liver cancer cells to the FDG/DPI combination (Fig. 4c). In the presence of FDG and DPI, at sub-optimal concentrations (lower than the concentrations used in previous experiments) that did not cause significant cell death either alone or in combination, the addition of PER to HK1^−^HK2^+^ liver cancer cells resulted in substantial lethality (Fig. 4d). A similar sensitizing effect was observed with another FAO inhibitor, etomoxir (Additional file 1: Figure S5B). While the FDG/DPI combination significantly decreased lipid droplets (lipid droplets are an indicator of cellular fatty acid abundance) in HK1^−^HK2^+^ liver cancer cells, PER addition prevented the disappearance of lipid droplets (Fig. 4e). In addition, PER synergized with the FDG/DPI combination to activate AMPKα, suppress the mTOR pathway, and trigger apoptosis (Fig. 4f). In contrast, there was no significant synthetic lethality induced by the triple combination of shHK2/DPI/PER in HK1^+^HK2^+^ cancer cells (Additional file 1: Figure S5C), suggesting that our shHK2/DPI/PER triple-combination treatment is selectively cytotoxic for HK1^−^HK2^+^ cancer cells and is likely to be tolerated by normal tissues. Taken together, our results indicate that FAO inhibition further sensitizes HK1^−^HK2^+^ cells to the combined inhibition of glycolysis and the mitochondrial electron transport chain (ETC) and that the triple combination of HK2 inhibition, DPI, and PER simultaneously target the components of multiple energy generation sources (glycolysis, OXPHOS, FAO) in HK1^−^HK2^+^ liver cancer cells, resulting in synthetic lethality in cells that express HK2 but not HK1.

### The HK2i/DPI/PER combination suppresses energy generation and alters cell metabolism in HK1^−^HK2^+^ liver cancer cells

**Fig. 5:**
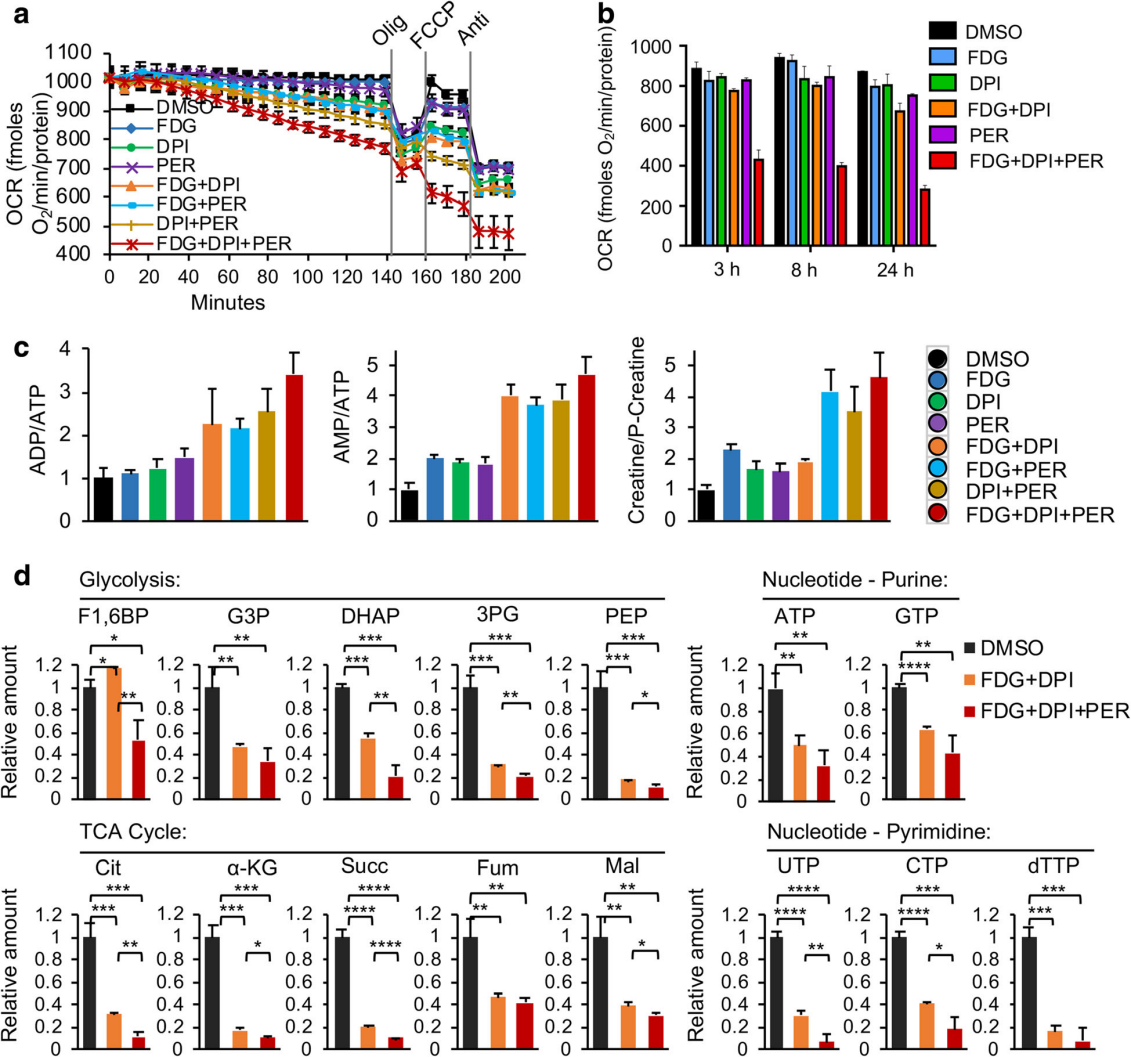
Modulation of energy generation and metabolism in HK1^−^HK2^+^ Hep3B/shHK2^DOX^ cells in response to FDG/DPI/PER treatment. **a** FDG/DPI/PER combination treatment acutely decreases the OCR of Hep3B cells. FDG (250 μM), DPI (15 nM), and/or PER (5 μM) were added to cultured Hep3B cells, as single agents or in the combinations shown, at time 0. The OCR was measured by the Seahorse assay. Oligomycin (2 μM), FCCP (0.1 μM), and Antimycin (2 μM) were used to indicate the fraction of respiration coupled to oxidative ATP production, maximum mitochondrial respiration, and non-mitochondrial respiration, respectively. **b** OCR in Hep3B cells after 3, 8, and 24 h of drug exposure; concentrations are as in panel **a**. **c** The HK2i/DPI/PER triple combination decreases liver cancer cellular energy levels. After an 8-h drug treatment, AMP, ADP, ATP, creatine, and P-creatine amounts in Hep3B cells were determined by LC-MS. **d** Changes in pool sizes of glycolysis and TCA cycle metabolites, as well as purine and pyrimidine nucleotides, after vehicle, FDG/DPI, or FDG/DPI/PER treatment in Hep3B cells. F1,6BP fructose 1,6-biphosphate, G3P glyceraldehyde 3-phosphate, DHAP dihydroxyacetone phosphate, 3PG 3-phospho-glycerate, PEP phosphoenolpyruvate, Cit citrate, α-KG α-ketoglutarate, Succ succinate, Fum fumarate, Mal malate. Each data point in **a** and **b** represents mean ± SEM of five samples, and each data point in **c** and **d** represents mean ± SD of triplicate samples. *P < 0.05, **P < 0.01, ***P < 0.001, ****P < 0.0001

Mitochondrial oxygen consumption rate (OCR) is an indicator of cellular energy production. To demonstrate a synergistic relationship among FDG, DPI, and PER, these inhibitors were used at sub-optimal concentrations (lower than the concentrations used in previous experiments), at which each individual agent did not substantially affect cell growth (Fig. 4c, d) or their OCR (Fig. 5a). The FDG/DPI/PER combination substantially reduced the basal OCR in HK1^−^HK2^+^ Hep3B cells within the first 2 h (Fig. 5a). PER enhanced the FDG/DPI combination-induced OCR reduction continued over a 24-h period (Fig. 5b). Decreased basal respiration in response to the triple combination suggests decreased ATP turnover in these cells, reflecting a state of reduced cellular energy generation [27].

Mass spectrometry was used to determine the levels of energy-related molecules extracted from HK1^−^HK2^+^ Hep3B cells after an 8-h treatment with FDG, DPI, and/or PER at sub-optimal concentrations, at which each individual agent did not substantially affect cell growth (Fig. 4c, d) or OCR (Fig. 5a). The increase in the ADP/ATP and AMP/ATP ratios in response to the triple treatment demonstrated the combined inhibition of ATP production from glycolysis, oxidative phosphorylation, and FAO (Fig. 5c). In addition, the combination of PER with FDG, DPI, or FDG/DPI substantially increased the creatine/P-creatine ratio (Fig. 5c), limiting the ability of HK1^−^HK2^+^ liver cancer cells to replenish ATP from P-creatine. These molecular energy changes, measured after an 8-h treatment, occurred before cell death as measured by trypan blue exclusion (Additional file 1: Figure S6A). The FDG/DPI/PER-induced reduction in ATP was also confirmed in living Hep3B cells expressing firefly luciferase, an ATP-dependent reporter enzyme (Additional file 1: Figure S6B). Metabolomic analysis also revealed that the pools of most metabolites of glycolysis and the TCA cycle, as well as nucleotide pools, were reduced in HK1^−^HK2^+^ liver cancer cells treated with FDG/DPI and were further decreased when PER was added into the combination (Fig. 5d). These data suggest that global changes in cell metabolism, in association with or caused by energy inhibition, may contribute to the HK2i/DPI/PER-induced synthetic lethality in HK1^−^HK2^+^ cancer cells.

### The shHK2/DPI/PER combination suppresses established HK1^−^HK2^+^ liver tumor progression in vivo

**Fig. 6:**
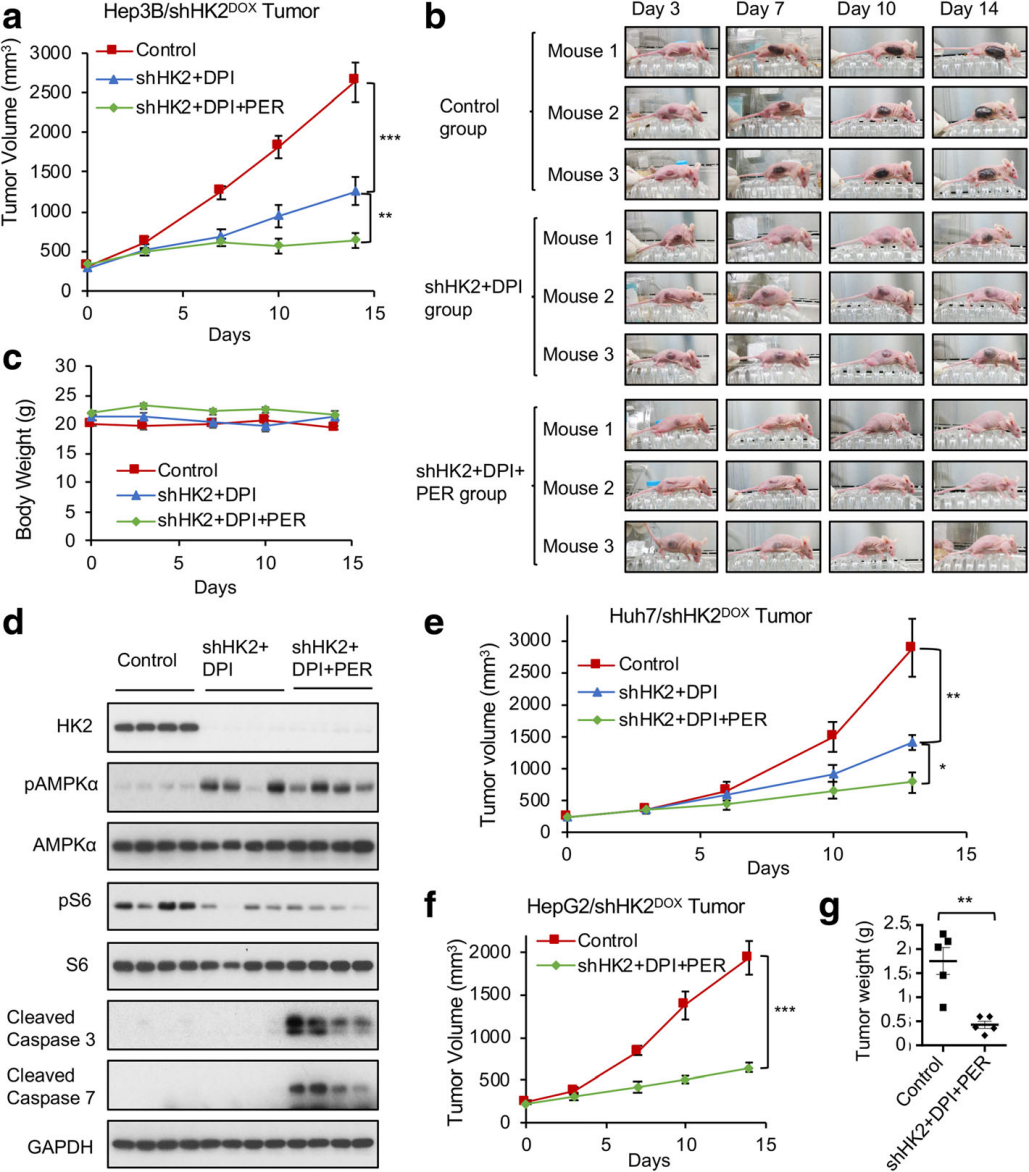
PER sensitizes established xenograft HK1^−^HK2^+^ liver tumors to HK2 knockdown/DPI combination therapy. **a** PER enhances the ability of the HK2 knockdown/DPI combination to retard the progression of Hep3B/shHK2^DOX^ tumor xenografts. When tumors reached 200 mm^3^ (day 0), xenografts were randomized into a control group and two treatment groups. The control mice (*n* = 7) were remained on the standard diet and were treated with vehicle. In the treatment groups, mice were placed on a DOX-supplemented diet (from day 0) and treated either with DPI (2 mg/kg, daily i.p. from day 3, *n* = 7) or with [DPI (2 mg/kg, daily i.p.) + PER (30 mg/kg, daily i.p), from day 3, *n* = 8]. **b** Representative images of tumor progression in three mice from each group in panel **a**. **c** Body weights of the mice in panel **a** bearing xenograft subcutaneous tumors, in response to the indicated treatments. **d** shHK2/DPI/PER treatment activates AMPKα and dephosphorylates S6 and elicits cleavage of caspase-3 and caspase-7. Hep3B/shHK2^DOX^ tumors from the indicated treatment groups were collected at day 15. Protein extracts from tissue homogenate supernatants were analyzed. **e** PER enhances the ability of the HK2 knockdown/DPI combination to retard the progression of HK1^−^HK2^+^ Huh7shHK2^DOX^ liver tumor xenografts. Experimental conditions are the same as those described in panel **a** (*n* = 6). **f** PER enhances the ability of the HK2 knockdown/DPI combination to retard the progression of HK1^−^HK2^+^ HepG2/shHK2^DOX^ liver tumor xenografts. Experimental conditions are the same as those described in panel **a** (*n* = 5). **g** HK2 knockdown/DPI/PER combination suppresses HepG2/shHK2^DOX^ tumor growth. Weights of HepG2/shHK2^DOX^ tumors after indicated treatments of the xenograft-bearing mice shown in panel **f** are shown. All data are expressed as means ± SEM. *P < 0.05. **P < 0.01. ***P < 0.001

We next compared the efficacy of the shHK2/DPI/PER triple combination to the efficacy of the shHK2/DPI double combination in xenograft HK1^−^HK2^+^ liver tumors. In each of the treatment groups, individual HK1^−^HK2^+^ Hep3B/shHK2^DOX^ mice were switched to the DOX-containing diet when their tumor reached 200 mm^3^ (day 0) to induce HK2 knockdown. DPI and/or PER treatments were started 72 h later (day 3). While PER alone showed no significant effects on tumor growth (Additional file 1: Figure S7), PER significantly enhanced the potency of the shHK2/DPI combination in Hep3B/shHK2^DOX^ tumor progression (Fig. 6a, b). No significant change in body weight was detected among the different Hep3B/shHK2^DOX^ xenograft groups (Fig. 6c). While both the shHK2 + DPI and the shHK2 + DPI + PER treatments activated AMPKα and suppressed the mTOR signaling cascade, only the shHK2 + DPI + PER treatment induced the appearance in xenograft tumors of the apoptosis markers cleaved caspase-3 and cleaved caspase-7 (Fig. 6d). These data indicate that, with the reported maximum DPI tolerated dosage and schedule [28], the intratumor amount of DPI was not sufficient to achieve tumor synthetic lethality with HK2 knockdown. However, the addition of PER sensitized Hep3B tumors to the shHK2/DPI combination and induced tumor cell apoptosis.

The potency of the shHK2/DPI/PER combination was also examined with two additional HK1^−^HK2^+^ liver tumor xenografts, Huh7/shHK2^DOX^ and HepG2/shHK2^DOX^ (Fig. 6e–g). These results confirm the in vivo efficacy and safety of our triple-combination therapy in established HK1^−^HK2^+^ liver tumors.

### The shHK2/DPI/PER combination is an effective precision therapy for treating HK1^−^HK2^+^ tumors, regardless of their tissues of origin

**Fig. 7:**
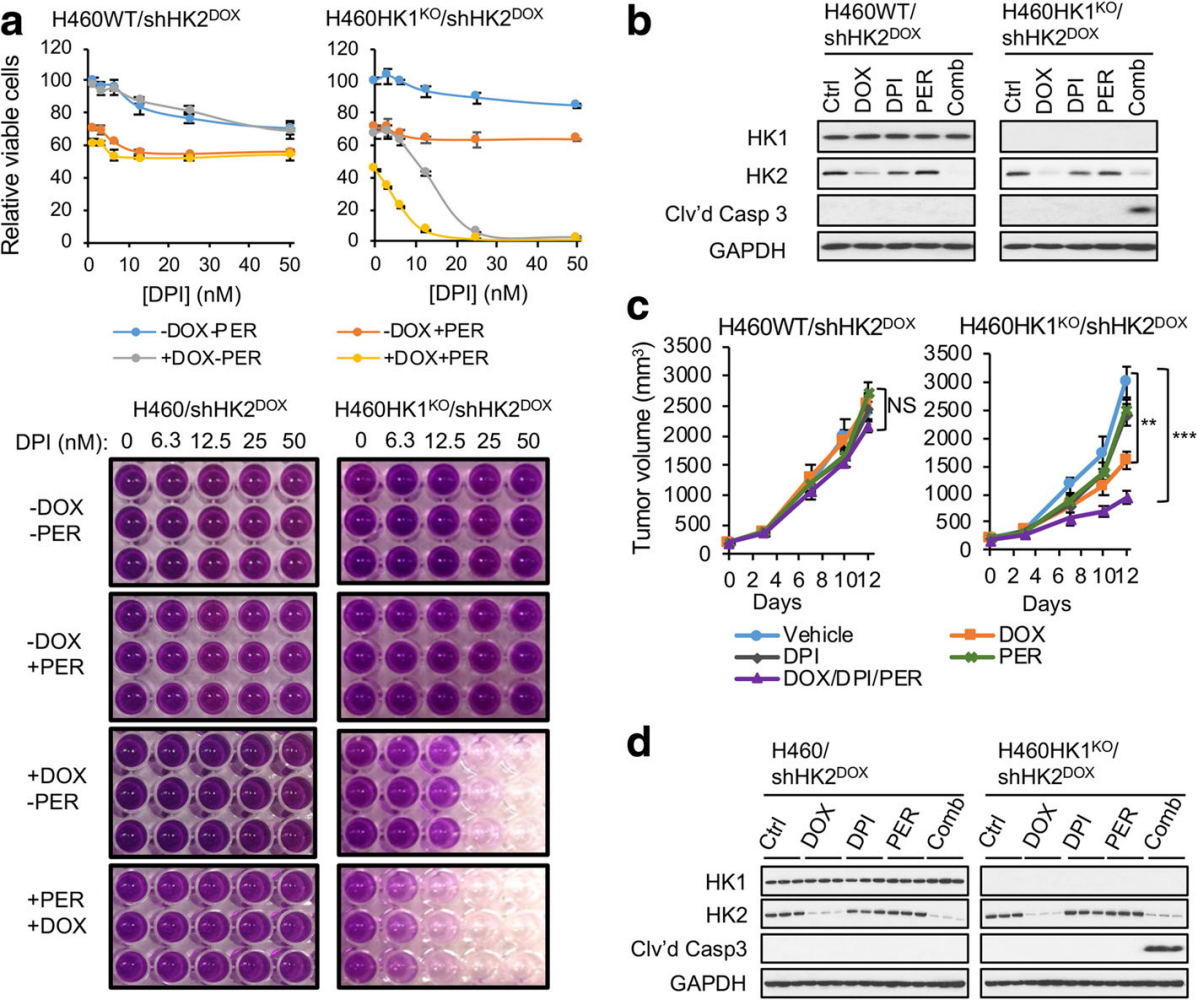
The shHK2/DPI/PER combination does not inhibit proliferation or tumor progression for HK1^+^HK2^+^ cancer cells but is effective in inhibiting proliferation and progression for the isogenic HK1^KO^HK2^+^ cancer cells. **a** Effect of the shHK2/DPI/PER combination on isogenic HK1^+^HK2^+^ H460WT/shHK2^DOX^ and H460HK1^KO^/shHK2^DOX^ cell proliferation. Cells were pretreated with vehicle or DOX for 2 days, followed by a 72-h treatment with DPI in the indicated concentration range, in the presence or absence of DOX and the presence or absence of PER (5 μM). Upper panels, MTT assay values were normalized to control samples (-DOX, -DPI, -PER) of the individual isogenic cell lines. Data are expressed as means ± SD. Lower panels, representative images of MTT assay results. **b** The shHK2/DPI/PER combination triggers apoptosis in H460HK1^KO^/shHK2^DOX^ cells but not in H460WT/shHK2^DOX^ cells. Cells were pretreated with vehicle or DOX for 2 days, followed by a 24-h treatment with 15 nM DPI, 5 μM PER, and DOX, both as single agents and in combination (Comb). Cell lysates were examined by Western blot for HK1, HK2, and cleaved caspase 3 expression. **c** The shHK2/DPI/PER combination inhibits the progression of H460HK1^KO^/shHK2^DOX^ tumor xenografts but not H460WT/shHK2^DOX^ tumor xenografts. When tumors reached 200 mm^3^ (day 0), xenografts were randomized into five groups (*n* = 5 per group) receiving vehicle, DOX in the diet, DPI (2 mg/kg, daily i.p.), and PER (30 mg/kg, daily i.p.), both as single agents and in combination. Data are expressed as means ± SEM. **P < 0.01. ***P < 0.001. NS not significant. **d** The shHK2/DPI/PER combination triggers apoptosis in H460HK1^KO^/shHK2^DOX^ tumor xenografts but not H460WT/shHK2^DOX^ tumor xenografts. Three representative xenograft tumors in each group in panel **c** were homogenized for Western blotting analyses of indicated proteins

We used liver cancer cell lines to develop our HK1^−^HK2^+^ cancer cell-targeted combination therapy. As indicated earlier, analysis of the CCLE RNASeq dataset identified subsets of cancer cell lines, present in a broad spectrum of cancers from a wide variety of tissues of origin, that exhibit the HK1^−^HK2^+^ molecular signature (Fig. 1d). We suggest that this therapy will be effective in HK1^−^HK2^+^ cancer cells, regardless of their tissue of origin. However, HK1^+^HK2^+^ cancer cell lines from a variety of cancers (liver, breast, colon, prostate, ovary, lung), including the lung cancer H460 cell line, are resistant to HK2 silencing alone (Fig. 2a–d) or the synthetic lethality of the shHK2/DPI/PER (Additional file 1: Figure S5C). To test the potential generality of the efficacy of the shHK2/DPI/PER combination in cancers with the HK1^−^HK2^+^ molecular characteristic, regardless of their tissues of origin, we used the isogenic lung cancer cell line pair, HK1^KO^HK2^+^ H460 cells created by CRISPR Cas9 knockout, and their parental HK1^+^HK2^+^ H460 cells to establish HK1^KO^HK2^+^/shHK2^DOX^ H460 cells and HK1^+^HK2^+^/shHK2^DOX^ H460 cells (Additional file 1: Figure S1C). Unlike the HK1-expressing HK1^+^HK2^+^/shHK2^DOX^ H460 cells, the isogenic HK1^KO^HK2^+^/shHK2^DOX^ H460 cells are sensitive to the synthetic lethality of the shHK2/DPI/PER combination in cell culture (Fig. 7a). The shHK2/DPI/PER combination triggers the cleavage of caspase 3 in the isogenic HK1^KO^HK2^+^ shHK2^DOX^ H460 cells but not in the HK1^+^HK2^+^shHK2^DOX^ H460 cells (Fig. 7b). While shHK2, DPI, or PER had no significant effect on xenograft HK1^+^HK2^+^ shHK2^DOX^ H460 tumor growth, shHK2 alone reduced tumor volume by 47%, and the shHK2/DPI/PER combination reduced tumor volume by 69% in HK1^KO^HK2^+^ shHK2^DOX^ H460 tumors (Fig. 7c). In addition to growth suppression in HK1^KO^HK2^+^ shHK2^DOX^ H460 tumors, the shHK2/DPI/PER combination also triggered tumor cell apoptosis (Fig. 7d). Taken together, our findings suggest that the HK2 inhibition/DPI/PER combination is a potential precision therapeutic approach to treatments for cancers with the HK1^−^HK2^+^ characteristic existing in a broad spectrum of cancer types, regardless of their tissues of origin.

## Discussion

In this study, we describe a triple-combination therapy for treatment of HK1^−^HK2^+^ cancers that targets the HK1^−^HK2^+^ cancer-specific modes of energy generation, HK2-driven glycolysis, mitochondrial oxidative phosphorylation, and fatty acid oxidation. Using HK1^−^HK2^+^ liver cancer cells as an example, we illustrated that targeting each individual energy generation source is either cytostatic or has only a subtle effect on cell proliferation or tumor progression for HK1^−^HK2^+^ liver cancer cells. While the majority of cancers including liver cancer, from many tissues of origin, express both HK1 and HK2, in many types of cancers, there exist subsets of HK1^−^HK2^+^ tumors, i.e., tumors that express only HK2. The potential specificity of this therapy for this HK1^−^HK2^+^ subclass, and the extension of this therapy across cancers from a broad range of tissues of origin, results from this common phenotype that makes them vulnerable to this combination therapy.

We have studied HK2 knockdown in a large panel of naturally existing HK1^+^HK2^+^ and HK1^−^HK2^+^ cancer cells and found that only HK1^−^HK2^+^ cancer cells but not HK1^+^HK2^+^ cells as defined by mRNA and Western blot analyses are sensitive to HK2 knockdown. It is likely that there is a threshold effect for HK1 expression necessary to provide resistance to HK2 inhibition. Future studies in the titration of HK1 protein levels using genetic manipulation approaches, such as inducible knockdown of HK1 in HK1^+^HK2^+^ cancer cells with different concentrations of doxycycline or inducible expression of HK1 in HK1^−^HK2^+^ cancer cells with different concentrations of doxycycline, would quantify the threshold effect of HK1 required to provide resistance to HK2 inhibition in cancer cells. In addition, how the HK1 gene is silenced in the HK1^−^HK2^+^ subsets of cancers is unknown. Future studies in regulation of HK1 expression in cancers and in normal tissues may provide targets for combination therapies in conjunction with HK2 inhibition for cancer treatment.

It is important to note that, even at the highest DPI concentration (100 nM) we used in cell culture experiments, which is relevant to the tolerated plasma concentration previously determined in mice [28], DPI inhibits only 50% of OXPHOS activity (Fig. 3d). These data suggest that the residual OXPHOS system can still oxidize fuels provided by metabolic pathways such as pyruvate oxidation, glutamine catabolism, and FAO. When we partially inhibited HK2-driven glycolysis and OXPHOS in HK1^−^HK2^+^ cancer cells, we observed that FAO was upregulated as indicated by ACC phosphorylation (Fig. 4a) and disappearance of intracellular lipid droplets (Fig. 4e). Therefore, we targeted FAO with the clinical FAO inhibitor PER. The addition of PER sensitized HK1^−^HK2^+^ cancer cells to the combination of FDG and DPI, indicating that FAO plays an important role in providing fuels to the residual OXPHOS activity, among the different fuel-providing pathways.

Because cancer cells can flexibly reprogram their energy generation [29], loss of HK2 activity in HK1^−^HK2^+^ cancer cells is only cytostatic; however, the combined inhibition of these three critical pathways involved in ATP production leads to a profound decrease in energy generation and to synthetic lethality for HK1^−^HK2^+^ cancers. Identification/stratification of patients with an HK1^−^HK2^+^ cancer molecular characteristic will be essential for clinical translation of our combination therapy as a precision medicine for this tumor subtype in tumors of different origins.

While our manuscript was in preparation for submission, De Waal et al. [11] reported that cell proliferation and xenograft progression of HepG2 and Huh7 liver cancer cells, which are from the HK1^−^HK2^+^ liver cancer subset we studied (Fig. 1e), are restricted by shHK2^DOX^ expression and that combination of shHK2^DOX^ expression and OXPHOS inhibition with metformin further suppressed Huh7 xenograft progression. We also find shHK2^DOX^ HK2 inhibition plus OXPHOS inhibition (in our case with DPI) reduces, but does not completely suppress, xenograft tumor progression for Huh7 (Fig. 6e) and Hep3B (Fig. 6a) HK1^−^HK2^+^ liver cancer xenografts. We suggest inhibition of the third major pathway driving ATP production, fatty acid oxidation, would enhance inhibition (by shHK2^DOX^ + DPI/metformin) of HK1^−^HK2^+^ liver tumor xenograft growth and confirm this hypothesis for Hep3B, Huh7, and HepG2 liver tumor xenograft growth (Fig. 6a, e, and f).

De Waal et al. [11] emphasized that many liver cancers express HK2; however, a substantial proportion of liver cancers express both HK1 and HK2 or only HK1 (Fig. 1c). We find neither cell proliferation (Fig. 2a) nor colony formation (Fig. 2b) are affected by shHK2^DOX^ HK2 inhibition for HK1^+^HK2^+^ HLF and JHH6 liver cancer cells, suggesting pharmacologic HK2 inhibition will not have therapeutic efficacy for HK1^+^HK2^+^ liver cancers.

CCLE cell gene expression profile analyses revealed cancers from tissues of multiple origins contain HK1^−^HK2^+^ subsets (Fig. 1d). To determine whether HK1^−^HK2^+^ cancers of other origins will be sensitive to HK2 inhibition, we created isogenic HK1^−^HK2^+^ cells from HK1^+^HK2^+^ H460 lung cancer cells. As expected, xenograft progression of HK1^+^HK2^+^ H460 cells was unaffected by shHK2^DOX^ expression or the shHK2^DOX^/DPI/PER combination (Fig. 7c). In contrast, HK1^KO^HK2^+^ H460 xenograft progression was inhibited by shHK2^DOX^ expression or shHK2^DOX^/DPI/PER (Fig. 7c). We conclude (i) that inhibition of HK2-driven glycolysis, OXPHOS, and FAO is likely to be a pan-tumor precision therapy approach to HK1^−^HK2^+^ tumors, regardless of their tissue of origin, and (ii) that cancer therapies that involve HK2 inhibition will be restricted to tumors that do not express HK1.

In addition to the hexokinases, several isoform switches in other metabolic enzymes occur during hepatocarcinogenesis, e.g., a splicing switch from ketohexokinase-C (KHK-C) to KHK-A involved in fructose metabolism [30], and a switch from 11β-HSD1 to 11β-HSD2 involved in glucocorticoid metabolism and gluconeogenesis [31]. Although the mechanisms of these metabolic isozyme switches and possible relationships among these switches during hepatocarcinogenesis are not currently understood, these additional cancer-specific isoforms may also be therapeutic targets for the development of effective cancer treatments.

Tumor progression for HK1^−^HK2^+^ liver cancer cells either by shHK2^DOX^ + metformin [11] or by shHK2^DOX^ + DPI (Fig. 3) is retarded but not completely suppressed. In vivo, DPI is maximally tolerated by mice at 2 mg/kg, with a plasma C_max_ value of 1 μM [28]. The combination of shRNA HK2 knockdown and daily DPI i.p. injection at 2 mg/kg did not cause HK1^−^HK2^+^ liver tumor cell apoptosis (Fig. 6d), suggesting that DPI at this dosage, in combination with HK2 knockdown, is not sufficient to cause lethality of HK1^−^HK2^+^ tumor cells in vivo. However, including PER in vivo to further sensitize HK1^−^HK2^+^ cancer cells to the shHK2/DPI combination improved the therapeutic effect on HK1^−^HK2^+^ tumors from growth inhibition to tumor cell death. Targeting fatty acid oxidation has recently been attracting additional attention for cancer therapy [13, 32, 33].

ATP is the principal energy currency in cell metabolism and is a versatile regulator of cellular activities [34, 35]. Maintenance of an adequate ATP supply is of crucial importance for cellular functions, for both normal tissues and for cancers. Targeting HK2-mediated glycolysis, mitochondrial respiration, and fatty acid oxidation in liver HK1^−^HK2^+^ cancer cells results in a synergistic decrease in cancer energy/ATP production. While a substantial drop in cellular energy levels causes HK1^−^HK2^+^ cancer cell death, the inhibition of different ATP generation sources may also have a profound impact on the distribution of compartmentalized intracellular ATP concentrations (“ATP inhomogeneity”) [36]. For example, ATP produced from glycolysis is readily diffused in the cytosol, whereas transfer of respiration-derived ATP from mitochondrial to cytosol requires carrier proteins across mitochondrial membranes [37]. Nuclear ATP levels are compromised by inhibiting mitochondrial ATP production but not by inhibiting glycolysis [38]. The subcellular compartmentalization of ATP has important roles in multiple cellular activities, such as cell motility [36], muscle contraction [39], DNA repair [40], and chromatin remodeling [38]. Further study will be required to explore the impact of our combination therapy on the ATP inhomogeneity in HK1^−^HK2^+^ cancer cells derived from alternative tissues and the consequent effects on cell viability.

To translate our findings to clinical treatment for HK1^−^HK2^+^ cancers, several steps will be required: (1) shHK2^DOX^ HK2 inhibition needs to be replaced by a selective HK2 inhibitor. GlaxoSmithKline recently reported their development of HK2-preferental inhibitors [10]. (2) DPI stood out among all screened compounds as the best synergistic partner with HK2 knockdown in HK1^−^HK2^+^ liver cancer cells. Although DPI has been extensively tested in pre-clinical animal models [28, 41–44], its safety in human subjects is not known. Clinical drugs with ETC-targeting effects, such as metformin [45] and papaverine [46], will need to be examined for their abilities to replace DPI in the combination therapy. (3) Doses, schedules, and routes of administration of the therapeutic agents involved in the triple combination will need optimization; the dosages in this study for xenograft tumor analyses were based on the previously reported single-agent dosages in mouse studies [28, 47]. Because of the synergistic effects, the required dosage of each agent for optimal combination therapy may well be lower than those used in these initial studies.

## Conclusions

We identified the HK1^−^HK2^+^ cancer subsets existing among a wide variety of cancer types. HK2 is the only active HK isoform driving glycolysis in the HK1^−^HK2^+^ subsets of cancers. The combination therapy we have developed specifically reduced the cellular ATP level in these HK1^−^HK2^+^ cancer cells to a level incompatible with cell survival. In conclusion, we suggest that reduction of ATP levels to a level that will no longer support the survival of HK1^−^HK2^+^ cancer cells can be clinically exploited as a targeted, precision medicine for HK1^−^HK2^+^ tumors, regardless of their tissue of origin.

## Additional files

Additional file 1: **Figure S1.** HK1^−^HK2^+^ cancer cells are highly sensitive to HK2 knockdown-induced growth inhibition. **Figure S2.** DPI synergizes with HK2 knockdown or inhibition in HK1^−^HK2^+^ liver cancer cells. **Figure S3.** DPI synergizes with HK2 silencing/inhibition by targeting mitochondrial complex I in HK1^−^HK2^+^ liver cancer cells. **Figure S4.** HK isoform expression in Hep3B/shHK2^DOX^ xenograft tumors with DOX and/or DPI treatments. **Figure S5.** Inhibition of fatty acid oxidation sensitizes HK1^−^HK2^+^ liver cancer cells to the HK2 inhibition/DPI combination. **Figure S6.** Modulation of HK1^−^HK2^+^ liver cancer cellular metabolism by the HK2i/DPI/PER combination. **Figure S7.** PER as a single agent does not have a significantly detectable effect on growth of subcutaneous Hep3B/shHK2^DOX^ tumors. (PPTX 782 kb)

Additional file 2: **Table S1.** The list of 119 FDA-approved oncology drugs provided by the National Cancer Institute (NCI) tested for synergy with DOX treatment in Hep3B/shHK2^DOX^ cells. **Table S2.** Synergy between HK2 inhibition and DPI. (DOCX 44 kb)

## Abbreviations

CT: Computed tomography
DOX: Doxycycline
DPI: Diphenyleneiodonium
ETC: Electron transport chain
FAO: Fatty acid oxidation
FDG: Fluorodeoxyglucose
HK: Hexokinase
PER: Perhexiline
PET: Positron emission tomography
