# Supplementary material for: A precision therapeutic strategy for hexokinase 1-null, hexokinase 2-positive cancers
Source: Cancer Metab. 2018 Jun 28;6:7. doi: 10.1186/s40170-018-0181-8 (PMC6022704; doi:10.1186/s40170-018-0181-8)
Supplement: Supplementary file 1 — Figure S1. HK1−HK2+ cancer cells are highly sensitive to HK2 knockdown-induced growth inhibition. Figure S2. DPI synergizes with HK2 knockdown or inhibition in HK1−HK2+ liver cancer cells. Figure S3. DPI synergizes with HK2 silencing/inhibition by targeting mitochondrial complex I in HK1−HK2+ liver cancer cells. Figure S4. HK isoform expression in Hep3B/shHK2DOX xenograft tumors with DOX and/or DPI treatments. Figure S5. Inhibition of fatty acid oxidation sensitizes HK1−HK2+ liver cancer cells to the HK2 inhibition/DPI combination. Figure S6. Modulation of HK1−HK2+ liver cancer cellular metabolism by the HK2i/DPI/PER combination. Figure S7. PER as a single agent does not have a significantly detectable effect on growth of subcutaneous Hep3B/shHK2DOX tumors. (PPTX 782 kb) [file 40170_2018_181_MOESM1_ESM.pptx]

## Slide 1
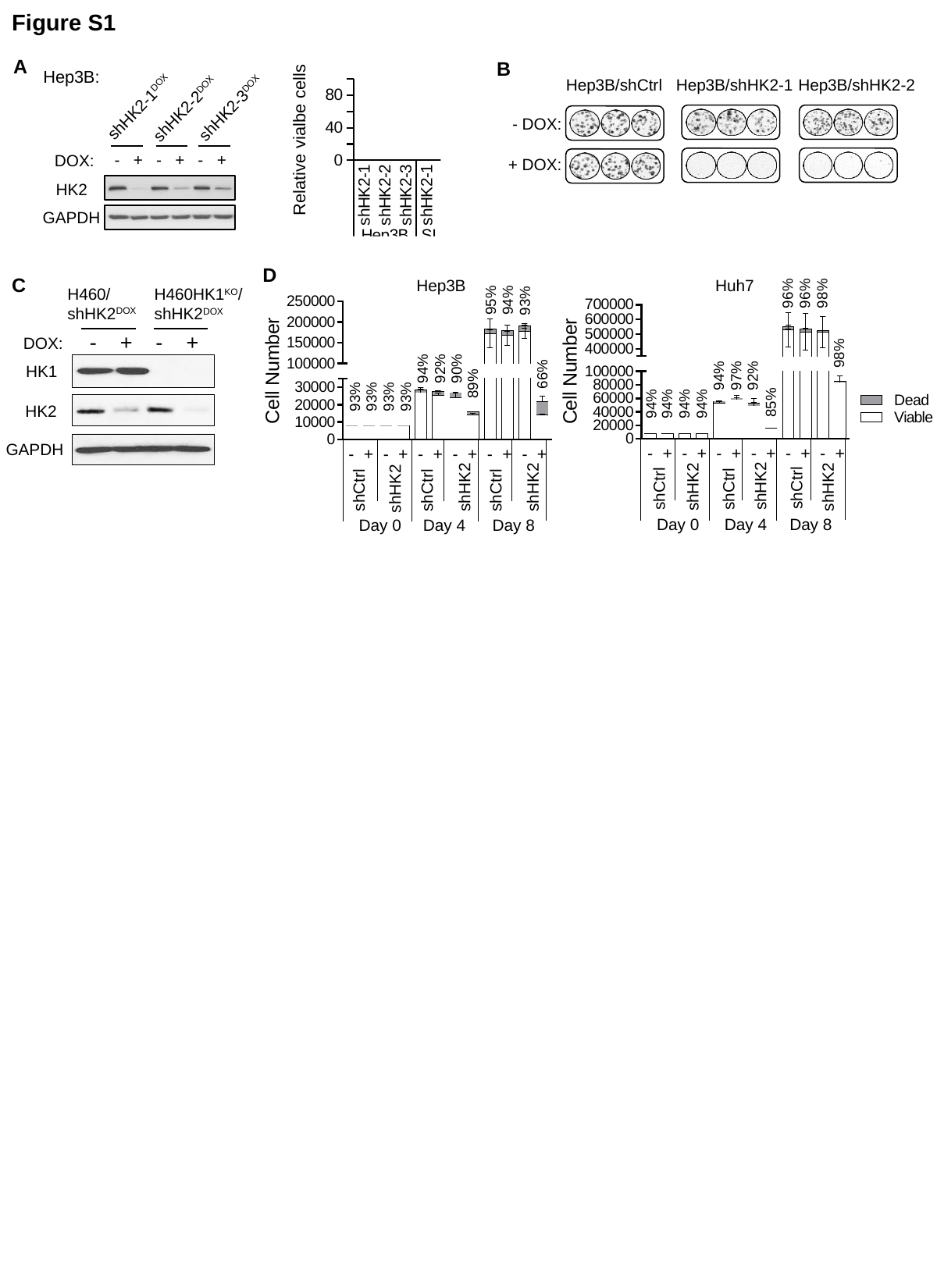

Figure S1
A
B
### Chart
| Category | |
|---|---|
| shHK2-1 | 29.09379968203498 |
| shHK2-2 | 41.52777777777778 |
| shHK2-3 | 74.26778242677825 |
| shHK2-1 | 90.42262085740349 |
| shHK2-2 | 90.60423916482125 |
| shHK2-3 | 95.35945226321796 |
| shHK2-1 | 104.9625468164794 |
| shHK2-2 | 93.27631966971394 |
| shHK2-3 | 98.49517319704712 |
Hep3B:
Hep3B/shCtrl
Hep3B/shHK2-1
Hep3B/shHK2-2
shHK2-2DOX
shHK2-3DOX
shHK2-1DOX
- DOX:
- + - + - +
DOX:
+ DOX:
HK2
GAPDH
D
C
Hep3B
Huh7
96%
96%
98%
H460/
shHK2DOX
H460HK1KO/
shHK2DOX
95%
94%
93%
- + - +
DOX:
98%
94%
92%
90%
HK1
66%
94%
97%
92%
89%
93%
93%
93%
93%
85%
94%
94%
94%
94%
HK2
GAPDH
shHK2
shHK2
shHK2
shCtrl
shCtrl
shCtrl
Day 0
Day 8
Day 4
shHK2
shHK2
shHK2
shCtrl
shCtrl
shCtrl
Day 0
Day 4
Day 8

## Slide 2
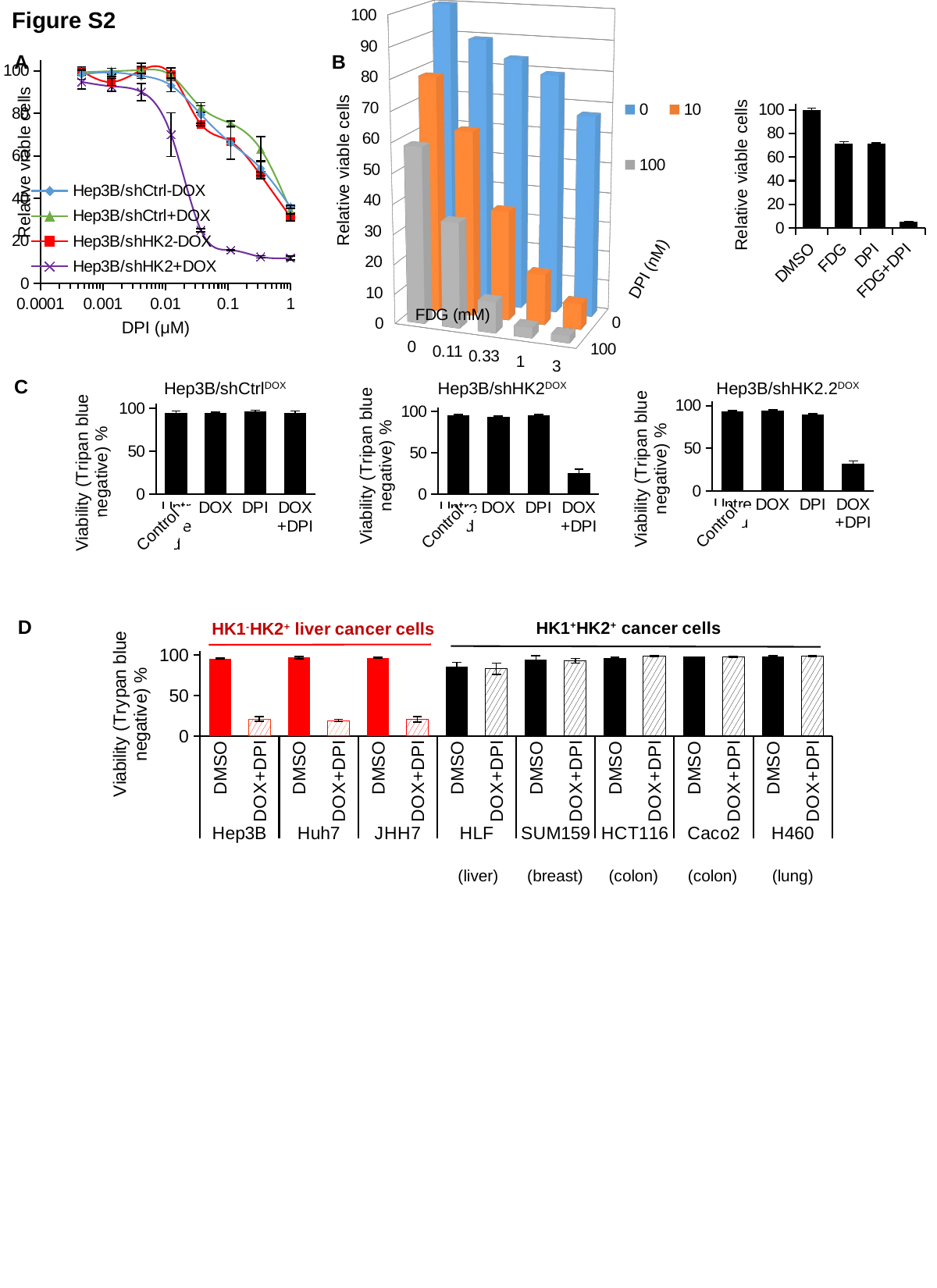

[unsupported chart]
Figure S2
### Chart
| Category | | | | |
|---|---|---|---|---|A
B
### Chart
| Category | |
|---|---|
| DMSO | 100.0 |
| FDG | 71.54285714285714 |
| DPI | 71.19999999999999 |
| FDG+DPI | 5.371428571428574 |DPI (nM)
### Chart
| Category | |
|---|---|
| Untreated | 95.5 |
| DOX | 93.0 |
| DPI | 95.5 |
| DOX+DPI | 26.0 |
### Chart
| Category | |
|---|---|
| Untreated | 93.5 |
| DOX | 94.5 |
| DPI | 89.5 |
| DOX+DPI | 32.0 |
### Chart
| Category | |
|---|---|
| Untreated | 94.0 |
| DOX | 94.5 |
| DPI | 96.0 |
| DOX+DPI | 94.0 |DPI (μM)
C
Hep3B/shCtrlDOX
Hep3B/shHK2DOX
Hep3B/shHK2.2DOX
Control
Control
Control
### Chart
| Category | |
|---|---|
| DMSO | 95.5 |
| DOX+DPI | 21.0 |
| DMSO | 97.0 |
| DOX+DPI | 19.0 |
| DMSO | 96.5 |
| DOX+DPI | 20.5 |
| DMSO | 86.0 |
| DOX+DPI | 83.0 |
| DMSO | 94.5 |
| DOX+DPI | 93.0 |
| DMSO | 96.5 |
| DOX+DPI | 98.5 |
| DMSO | 98.0 |
| DOX+DPI | 97.5 |
| DMSO | 98.5 |
| DOX+DPI | 98.5 |D
(liver)
(breast)
(colon)
(colon)
(lung)

## Slide 3
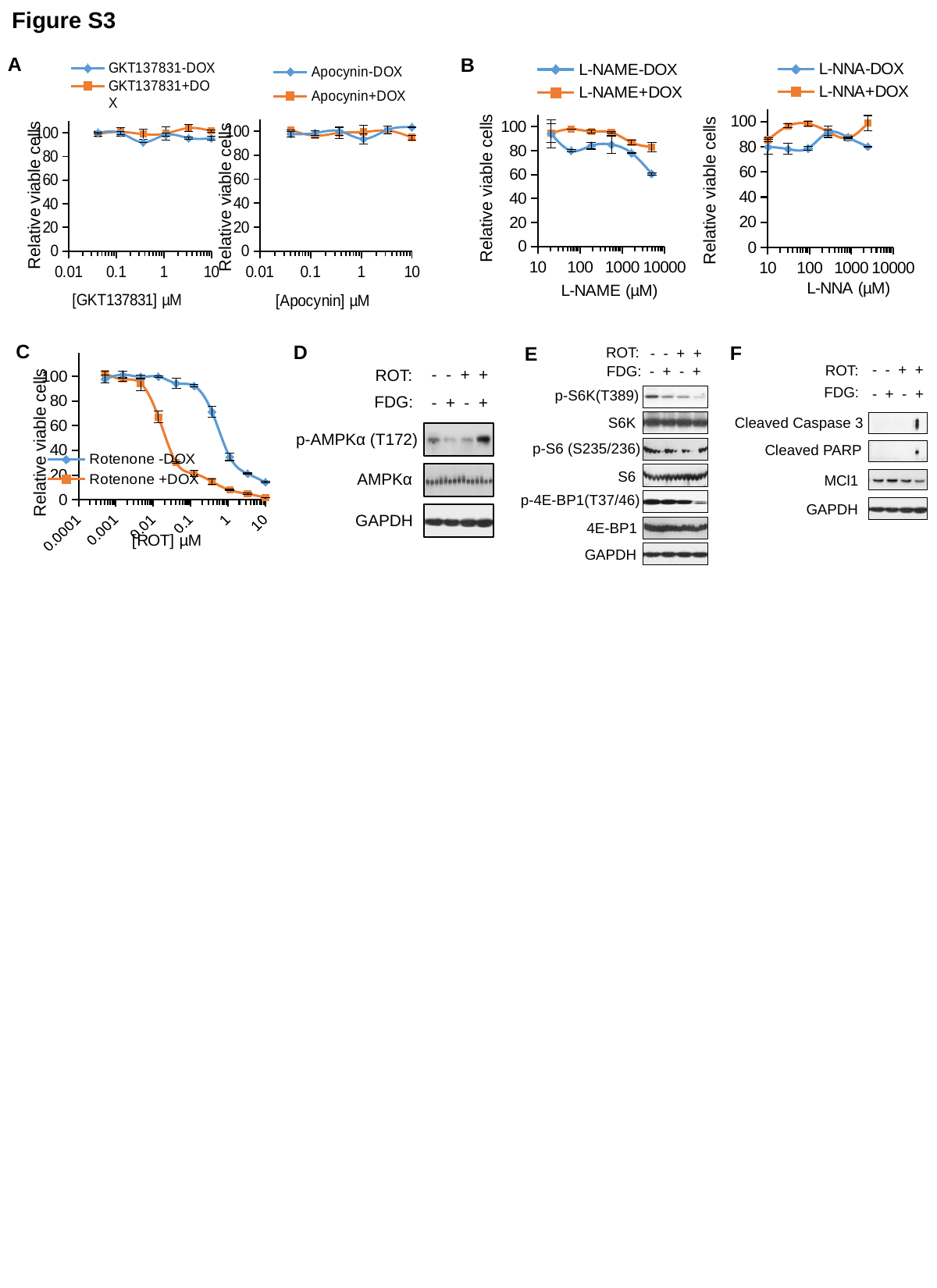

Figure S3
### Chart
| Category | L-NNA-DOX | L-NNA+DOX |
|---|---|---|
### Chart
| Category | L-NAME-DOX | L-NAME+DOX |
|---|---|---|A
B
### Chart
| Category | | |
|---|---|---|
### Chart
| Category | | |
|---|---|---|Relative viable cells
Relative viable cells
Relative viable cells
Relative viable cells
### Chart
| Category | Rotenone -DOX | Rotenone +DOX |
|---|---|---|C
D
F
E
ROT:
- - + +
- + - +
FDG:
p-S6K(T389)
S6K
p-S6 (S235/236)
S6
p-4E-BP1(T37/46)
4E-BP1
GAPDH
- - + +
ROT:
- - + +
ROT:
FDG:
- + - +
p-AMPKα (T172)
AMPKα
GAPDH
FDG:
- + - +
Cleaved Caspase 3
Relative viable cells
Cleaved PARP
MCl1
GAPDH

## Slide 4
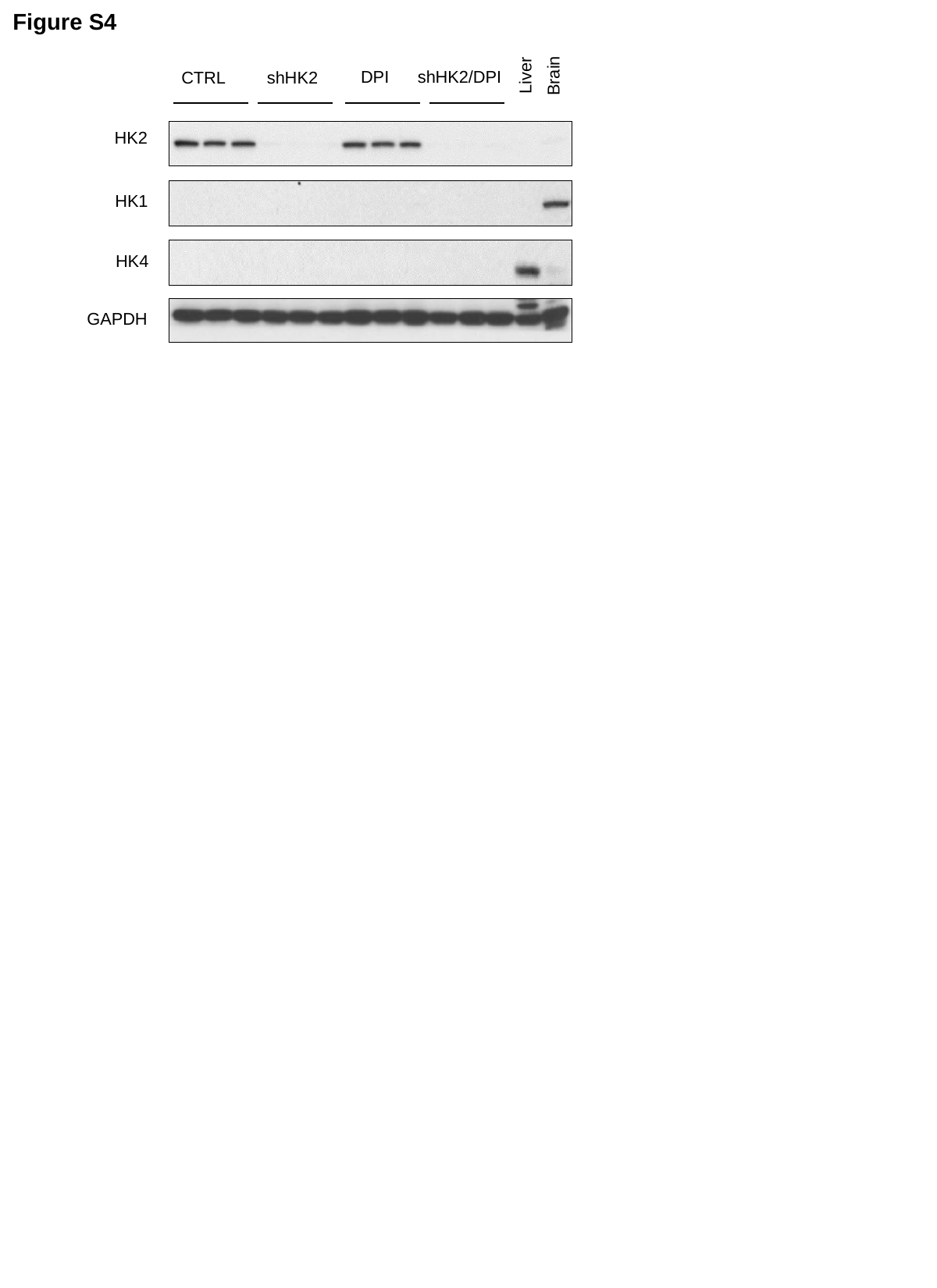

Figure S4
Liver
Brain
DPI
shHK2/DPI
CTRL
shHK2
HK2
HK1
HK4
GAPDH

## Slide 5
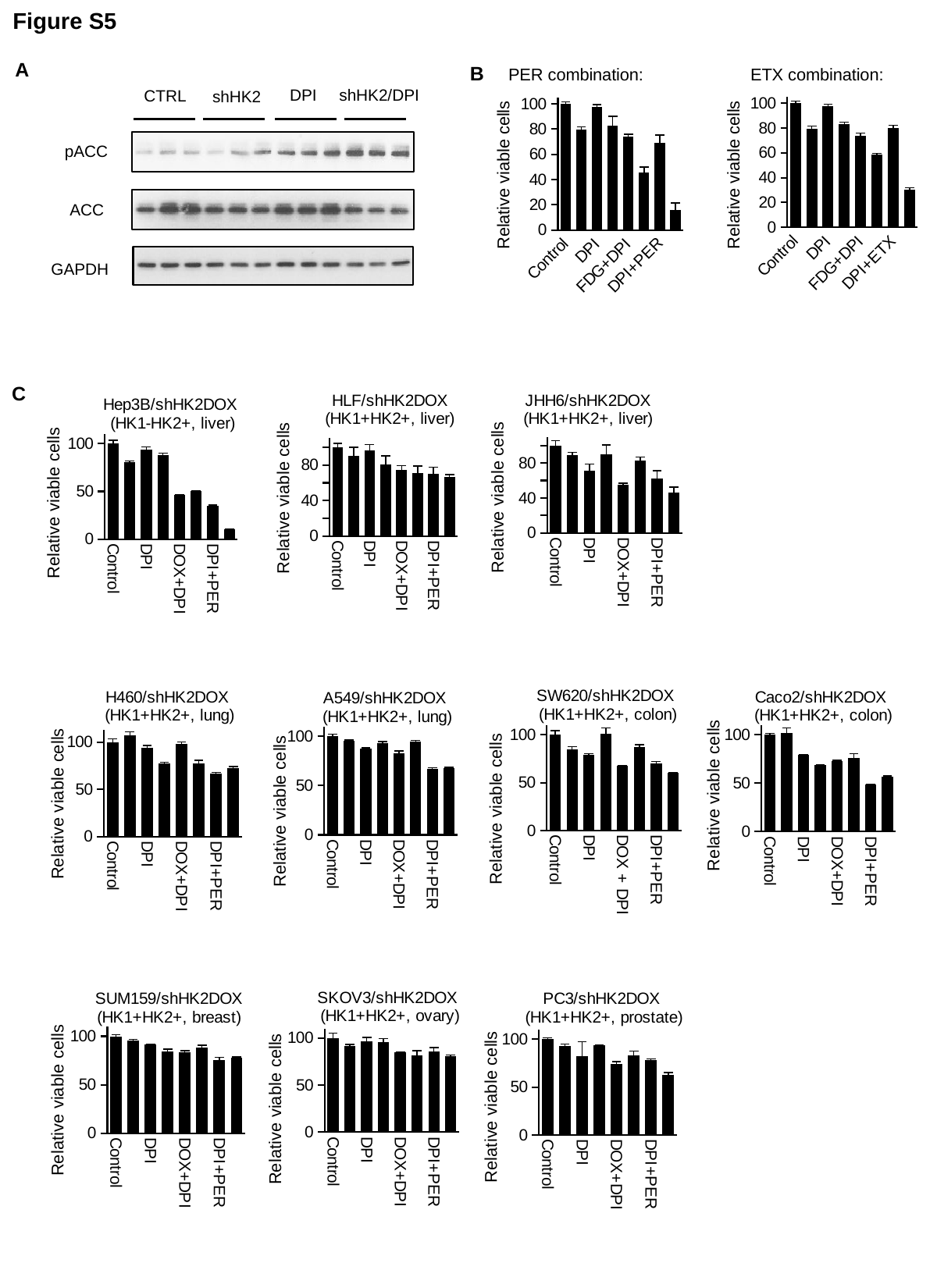

Figure S5
A
B
PER combination:
ETX combination:
### Chart
| Category | |
|---|---|
| Control | 99.99994249572022 |
| FDG | 79.70093174184488 |
| DPI | 97.58476274596735 |
| ETX | 83.09368424745013 |
| FDG+DPI | 73.95050376624279 |
| FDG+ETX | 58.596861071385234 |
| DPI+ETX | 79.81594030135693 |
| FDG+DPI+ETX | 30.083702609551946 |
### Chart
| Category | |
|---|---|
| Control | 99.99994249572022 |
| FDG | 79.70093174184488 |
| DPI | 97.58476274596735 |
| PER | 82.74865856891398 |
| FDG+DPI | 73.95050376624279 |
| FDG+PER | 45.830910965548604 |
| DPI+PER | 69.06263998698103 |
| FDG+DPI+PER | 15.657311669128509 |DPI
shHK2/DPI
CTRL
shHK2
pACC
Relative viable cells
Relative viable cells
ACC
GAPDH
C
### Chart: JHH6/shHK2DOX
(HK1+HK2+, liver)
| Category | |
|---|---|
| Control | 100.0 |
| DOX | 88.68184568047148 |
| DPI | 71.25389124155146 |
| PER | 89.58329159972597 |
| DOX+DPI | 55.42850732575052 |
| DOX+PER | 83.17300950724963 |
| DPI+PER | 62.439753364396495 |
| DOX+DPI+PER | 45.81308418703602 |
### Chart: HLF/shHK2DOX
(HK1+HK2+, liver)
| Category | |
|---|---|
| Control | 100.0 |
| DOX | 90.1102423786345 |
| DPI | 96.64657643873238 |
| PER | 81.01621238197656 |
| DOX+DPI | 74.2525275719622 |
| DOX+PER | 70.72859094825725 |
| DPI+PER | 70.38756482338259 |
| DOX+DPI+PER | 66.7499528247194 |
### Chart: Hep3B/shHK2DOX
(HK1-HK2+, liver)
| Category | Hep3B |
|---|---|
| Control | 100.00006024100014 |
| DOX | 80.66269919439712 |
| DPI | 93.67475522575616 |
| PER | 88.19282421254472 |
| DOX+DPI | 46.20484711135368 |
| DOX+PER | 50.18075312093563 |
| DPI+PER | 34.5180930831886 |
| DOX+DPI+PER | 10.421693025116282 |
### Chart: SW620/shHK2DOX
(HK1+HK2+, colon)
| Category | |
|---|---|
| Control | 100.0 |
| DOX | 84.70350404312669 |
| DPI | 78.90835579514825 |
| PER | 101.33395494061685 |
| DOX + DPI | 68.05929919137466 |
| DOX + PER | 87.46630727762803 |
| DPI+PER | 70.2181341421373 |
| DOX+DPI+PER | 60.38382453735435 |
### Chart: Caco2/shHK2DOX
(HK1+HK2+, colon)
| Category | Caco2 |
|---|---|
| Control | 99.99999999999999 |
| DOX | 101.96969696969695 |
| DPI | 78.75757575757575 |
| PER | 68.39393939393939 |
| DOX+DPI | 72.5151515151515 |
| DOX+PER | 76.3030303030303 |
| DPI+PER | 48.454545454545446 |
| DOX+DPI+PER | 56.6060606060606 |
### Chart: H460/shHK2DOX
(HK1+HK2+, lung)
| Category | |
|---|---|
| Control | 100.0 |
| DOX | 107.33204134366925 |
| DPI | 94.15374677002585 |
| PER | 77.51937984496124 |
| DOX+DPI | 98.0297157622739 |
| DOX+PER | 77.61627906976743 |
| DPI+PER | 66.828165374677 |
| DOX+DPI+PER | 72.86821705426355 |
### Chart: A549/shHK2DOX
(HK1+HK2+, lung)
| Category | A549 |
|---|---|
| Control | 100.0 |
| DOX | 95.63744176196526 |
| DPI | 87.2511647606946 |
| PER | 93.30792037272342 |
| DOX+DPI | 83.18509106310886 |
| DOX+PER | 94.53621346886911 |
| DPI+PER | 67.25963574756457 |
| DOX+DPI+PER | 67.55612028801356 |
### Chart: SKOV3/shHK2DOX
(HK1+HK2+, ovary)
| Category | |
|---|---|
| Control | 100.0 |
| DOX | 91.61882893226178 |
| DPI | 96.55568312284731 |
| PER | 95.85460853626606 |
| DOX+DPI | 85.30424799081517 |
| DOX+PER | 81.40068886337544 |
| DPI+PER | 86.09319294217173 |
| DOX+DPI+PER | 80.72289156626505 |
### Chart: SUM159/shHK2DOX (HK1+HK2+, breast)
| Category | |
|---|---|
| Control | 100.0 |
| DOX | 95.31590413943354 |
| DPI | 91.17647058823529 |
| PER | 84.53159041394333 |
| DOX+DPI | 83.73275236020334 |
| DOX+PER | 88.6710239651416 |
| DPI+PER | 75.12708787218591 |
| DOX+DPI+PER | 78.21350762527231 |
### Chart: PC3/shHK2DOX
(HK1+HK2+, prostate)
| Category | |
|---|---|
| Control | 100.0 |
| DOX | 93.14372778059929 |
| DPI | 82.57998984255968 |
| PER | 93.64270276882925 |
| DOX+DPI | 74.4540375825292 |
| DOX+PER | 83.29101066531234 |
| DPI+PER | 78.11860691606236 |
| DOX+DPI+PER | 62.87128712871288 |

## Slide 6
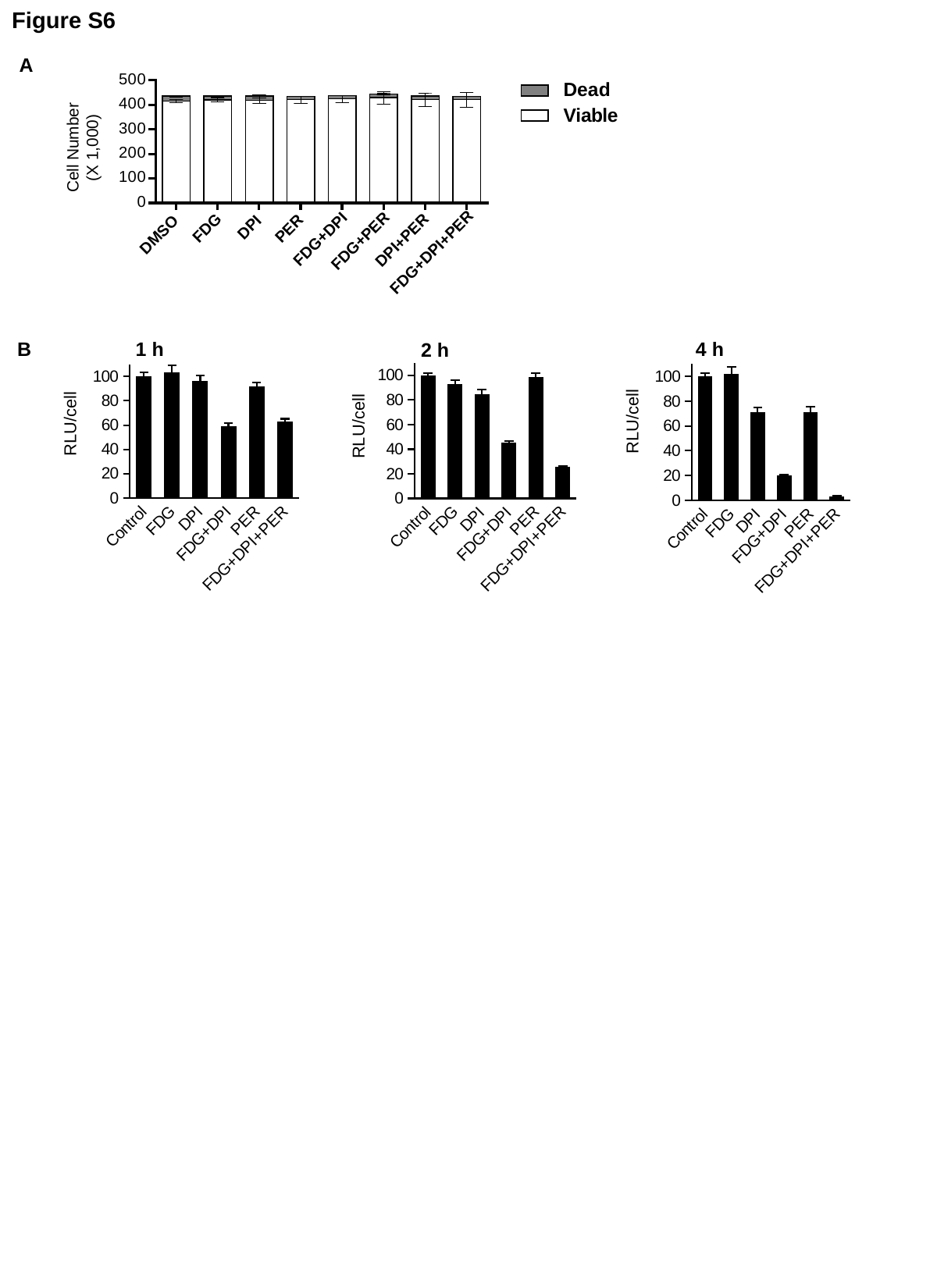

Figure S6
A
Cell Number
(X 1,000)
1 h
4 h
B
2 h
### Chart
| Category | |
|---|---|
| Control | 100.00000529107 |
| FDG | 102.14445110786095 |
| DPI | 70.93272505229301 |
| FDG+DPI | 20.152395991515455 |
| PER | 71.00658801372312 |
| FDG+DPI+PER | 2.8133160267937853 |
### Chart
| Category | |
|---|---|
| Control | 100.00001451534713 |
| FDG | 103.55029338021878 |
| DPI | 96.05250134832315 |
| FDG+DPI | 59.324070279735494 |
| PER | 92.09738934167146 |
| FDG+DPI+PER | 63.16027422243453 |
### Chart
| Category | |
|---|---|
| Control | 99.99998364866767 |
| FDG | 92.9499103555778 |
| DPI | 84.59018696342564 |
| FDG+DPI | 45.267804537736026 |
| PER | 98.48092260240911 |
| FDG+DPI+PER | 25.75877717168851 |

## Slide 7
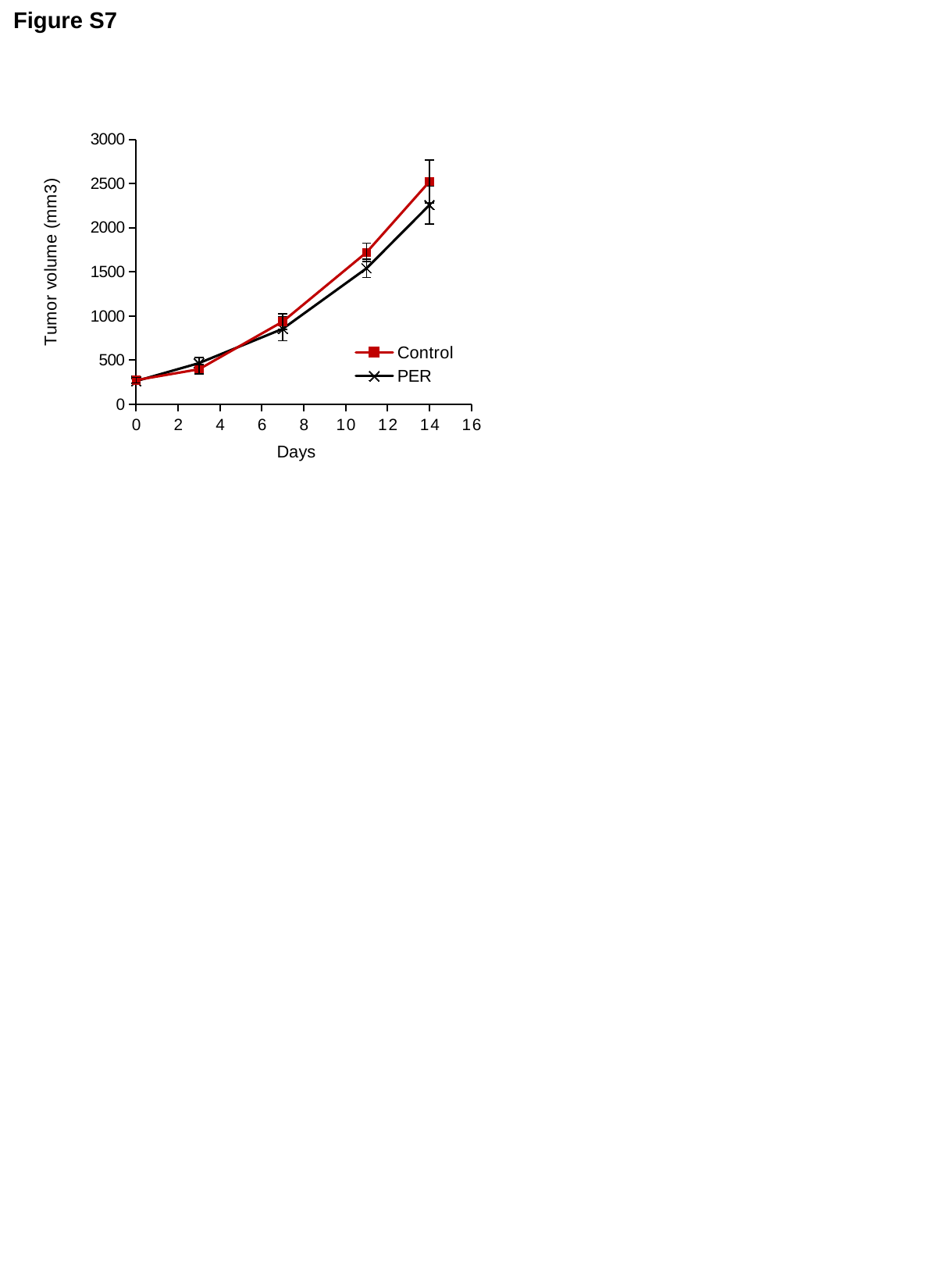

Figure S7
### Chart
| Category | Control | PER |
|---|---|---|
