# Supplementary material for: A precision therapeutic strategy for hexokinase 1-null, hexokinase 2-positive cancers
Source: Cancer Metab. 2018 Jun 28;6:7. doi: 10.1186/s40170-018-0181-8 (PMC6022704; doi:10.1186/s40170-018-0181-8)
Supplement: Supplementary file 2 — Table S1. The list of 119 FDA-approved oncology drugs provided by the National Cancer Institute (NCI) tested for synergy with DOX treatment in Hep3B/shHK2DOX cells. Table S2. Synergy between HK2 inhibition and DPI. (DOCX 44 kb) [file 40170_2018_181_MOESM2_ESM.docx]

**Additional file 2**

**A Precision Therapeutic Strategy for Hexokinase 1-Null, Hexokinase 2-Positive Cancers**

Shili Xu^1^, Arthur Catapang^1^, Daniel Braas^1,3^, Linsey Stiles^6^, Hanna M. Doh^1^, Jason T. Lee^1,4,5^, Thomas G. Graeber^1,3,4,5^, Robert Damoiseaux^1,7^, Orian Shirihai^6^, Harvey R. Herschman^1,2,4,5,8*^

^1^Department of Molecular and Medical Pharmacology

^2^Department of Biological Chemistry

^3^UCLA Metabolomics Center

^4^Crump Institute for Molecular Imaging

^5^Jonsson Comprehensive Cancer Center

^6^Division of Endocrinology, Department of Medicine

^7^California NanoSystems Institute

^8^Molecular Biology Institute

David Geffen School of Medicine, University of California, Los Angeles, Los Angeles, California 90095, USA

*Correspondence: hherschman@mednet.ucla.edu

**SUPPLEMENTAL METHODS**

**Antibodies**

HK1 (#2024), HK2 (#2867), p-S6K(T389) (#9234), S6K (#2708), p-S6(S235/236) (#4858), S6 (#2217), p-4EBP1(T37/46) (#2855), 4EBP1 (9452), cleaved Casp3 (#9664), cleaved PARP (5625), p-AMPKα(T172) (#2535), AMPKα (#5831), G6PD (#12263), p-ACC(S79) (#11818), and ACC (#3676) antibodies were from Cell Signaling Technology. HK4 antibody (#NBP1-81741) was from NOVUS. MCL1 antibody was from Abcam (#32087). GAPDH antibody (#sc-25778) was from Santa Cruz Biotechnology.

**Doxycycline (DOX)-Inducible shRNA Knockdown in Cultured Cells**

For inducible protein knockdown, shRNA oligonucleotides (shHK2-1: GGATGTGTGTGAACATGGAATTTCAAGAGAATTCCATGTTCACACACATCC; shHK2-2: CTTCATGGATAAGCTACAAATTTCAAGAGAATTTGTAGCTTATCCATGAAG; shHK2-3: CCAAAGACATCTCAGACATTGTTCAAGAGACAATGTCTGAGATGTCTTTGG; shScramble: TAGCGACTAAACACATCAATTCAAGAGATTGATGTGTTTAGTCGCTA; and shG6PD: GTCGGATACACACATATTCATTCAAGAGATGAATATGTGTGTATCCGAC) were annealed and ligated into pENTR/H1/TO vector (Invitrogen). The resulting shRNA constructs were recombined into pLentipuro3/BLOCK-iT-DEST, a gift from Dr. Andrew Aplin (Thomas Jefferson University), using Gateway LR Clonase II (Invitrogen). Recombinant lentiviruses were packaged in 293FT cells using the ViraPower Lentiviral Packaging Mix (Invitrogen). Lentivirus was harvested 72 h after transfection and used for infection. Cells were selected in 2 µg/mL puromycin for 10 days. DOX induction of knockdown is controlled by the Tet repressor (TetR) protein expressed from the pLenti0.3/EF/GW/IVS-Kozak-TetR-P2A-Bsd vector, which was constructed by Dr. Ethan Abel and was provide by Dr. Diane M. Simeone (University of Michigan, Translational Oncology Program). Knockdown was induced with 25 ng/ml DOX. Unless otherwise indicated, shHK2-1 was used in experiments for HK2 knockdown.

**MTT Assay**

The relative viable cell number was assessed using a 3-(4,5-dimethylthiazol-2-yl)-2,5-diphenyltetrazolium bromide (MTT) assay to evaluate cell growth and viability. Cells were seeded in 96-well microtiter plates, allowed to attach overnight and then exposed to indicated drugs for 72 h. At the end of drug treatments, the MTT solution was added to the wells (final concentration 0.5 mg/mL) and incubated for 3 h at 37°C. After removal of the supernatant, DMSO was added and absorbance was measured at 590 nm.

**Colony Formation Assay**

250 cells per well were seeded in 24-well plates 24 h before the indicated treatments. After 12-18 days, colonies were stained with 2% crystal violet solution.

**Seahorse Respirometry Assay**

Respirometry assays were performed on a Seahorse XF96 Extracellular Flux Analyzer. Hep3B cells were plated into an XF96 microplate at a final density of 13,000 cells per well. Cells, plated in growth medium, were maintained overnight in a cell culture incubator (37^◦^C; 5% CO_2_). Test compounds were either injected acutely from Port A during the respirometry assay or added to the cell plate 2, 7, or 23 hours prior to the start of the assay. Assay medium (Seahorse DMEM with 2 mM glutaMAX supplemented with 10 mM glucose, pH 7.4) was prepared on the day of the experiment. Hep3B cells were washed twice with assay medium and wells were brought to a final volume of 175 µL. Test compounds were added into the wells (except for compounds were injected during the assay) and were present during the assay. The XF96 microplate was placed in a 37^o^C incubator for 60 minutes prior to loading the plate into the instrument. The mitochondrial stress test conditions included injection of: the mitochondrial ATP Synthase inhibitor, oligomycin (final concentration of 2 μM); the chemical uncoupler, FCCP (final concentration of 0.1μM); and the complex III inhibitor, antimycin A (final concentration of 3 μM). Approximately 30 minutes prior to the end of the 1 hour incubation in assay medium, calibration of the XF96 Assay Cartridge commenced. Once the calibration process was completed, the plated cells were placed into the instrument to start the respirometry assay. Oxygen consumption rates were normalized to cellular protein content per well, which was measured from each well after completion of the respirometry assay.

**Western Blotting**

Cells were washed with ice-cold PBS, and lysed in cold lysis buffer (20 mM Tris-HCl, 150 mM NaCl, 1 mM EDTA, 1% Triton X-100, pH 7.5) with 1x protease and phosphatase inhibitors. Protein extracts (15 µg) were resolved on 10% SDS-PAGE and then electrotransferred to Immun-Blot PVDF membrane (Bio-Rad, Hercules, CA, USA). After blocking with 5% milk in TBS-T, membranes were probed with the indicated primary antibodies at 4 ̊C overnight, and then with horseradish peroxidase-conjugated secondary antibody at room temperature for 1 h. Blots were developed using Pierce ECL Substrate (Thermo Scientific, Rockford, IL) and exposed to X-ray films.

**Oil Red O Staining of Intracellular Lipid Droplets**

Cells were washed with PBS and fixed by 10% formalin for 10 min at room temperature. Fixed cells were washed with H_2_O and 60% isopropanol, and dried completely. Cells were then incubated in freshly prepared and filtered Oil Red O solution (0.3% w/v, Sigma) for 1 h at room temperature. Immediately after the Oil Red O solution was removed, cells were washed four times with H_2_O (Jiang et al., 2013).

**CRISPR Cas9 HK1 Knockout**

HK1 gRNA (AGATGTTGCCAACATTCGTA) was designed using the BROAD gRNA design tool, and ligated into the lentiCRISPRv2 vector. Lentiviruses carrying the lentiCRISPRv2-gRNA were packaged in 293FT cells using the ViraPower Lentiviral Packaging Mix (ThermoFisher), and harvested after 72 hr. Two days after lentivirus infection, target cancer cells were selected in 2 µg/mL puromycin for 7 days. After puromycin selection, cells were seeded in 96-well plates at one cell per well density, and allowed to grow into colonies. Single colonies were isolated and validated for HK1 knockout by Western blotting analysis of HK1 protein expression.

**Intracellular Metabolite Extraction and Analysis**

The experiments were performed as described (Krall et al., 2016). Briefly, cells were seeded in six-well plates, and metabolites were extracted at 70-80% confluence. After 8-hour exposure to the indicated treatments, cells were washed with ice-cold 150 mM ammonium acetate, and scraped off the plate in 800 µl ice-cold 80% methanol. 10 nmol norvaline was added as an internal standard. After vigorous vortexing, the samples were centrifuged at 12,000 rpm. Supernatants were transferred into glass vials and the metabolites were dried under vacuum. Metabolites were resuspended in 50 µl 70% acetonitrile (ACN), and 5 µl of this solution was used for the mass spectrometer-based analysis. The analysis was performed on a Q Exactive (Thermo Scientific) instrument, with polarity-switching (+3.50 kV/-3.50 kV) in full scan mode. Separation was achieved using A) 5 mM NH_4_AcO (pH 9.9) and B) ACN. The gradient started with 15% A) going to 90% A) over 18 min, followed by an isocratic step for 9 min and reversal to the initial 15% A) for 7 min. Metabolites were quantified with TraceFinder 3.3 using accurate mass measurements (≤ 3 ppm), as well as retention times and fragmentation patterns of purchased compounds.

***In Vivo* Assessment of Tumor Glucose Consumption by ^18^F-FDG PET/CT**

Animals were warmed on a heating pad for 30 min, anesthetized with 2% isoflurane in oxygen, and injected via the tail vein with 3.7 MBq of clinical-grade ^18^F-fluorodeoxyglucose (^18^F-FDG). Animals were maintained under anesthesia in a heated induction chamber during the 1-h ^18^F-FDG biodistribution period prior to imaging. Positron emission tomography (PET) and computed tomography (CT) scans were conducted on a G8 combined PET/CT instrument (Sofie Biosciences, Inc.) with a 600-s PET acquisition, and maximum-likelihood expectation maximization reconstruction, and with a 50-s CT acquisition and Feldkamp reconstruction. PET/CT images were co-registered and analyzed using A Medical Image Data Examiner (AMIDE) software version 1.0.4 (Loening and Gambhir, 2003).

**SUPPLEMENTARY REFERENCES**

Jiang, P., Du, W., Mancuso, A., Wellen, K. E., and Yang, X. (2013). Reciprocal regulation of p53 and malic enzymes modulates metabolism and senescence. Nature *493*, 689-693.

Krall, A. S., Xu, S., Graeber, T. G., Braas, D., and Christofk, H. R. (2016). Asparagine promotes cancer cell proliferation through use as an amino acid exchange factor. Nat Commun *7*, 11457.

Loening, A. M., and Gambhir, S. S. (2003). AMIDE: a free software tool for multimodality medical image analysis. Mol. Imaging *2*, 131-137.

**Table S1.** The list of 119 FDA-approved oncology drugs provided by the National Cancer Institute (NCI) tested for synergy with DOX treatment in Hep3B/shHK2^DOX^ cells.

|  | **Drugs** |  |  | **Drugs** |
| --- | --- | --- | --- | --- |
| 1 | Hydroxyurea |  | 31 | Thioguanine |
| 2 | Dacarbazine |  | 32 | Busulfan |
| 3 | Azacitidine |  | 33 | Cyclophosphamide |
| 4 | Streptozocin |  | 34 | Cisplatin |
| 5 | Vorinostat |  | 35 | Letrozole |
| 6 | Mitomycin |  | 36 | Belinostat |
| 7 | Fludarabine phosphate |  | 37 | Sunitinib |
| 8 | Erlotinib hydrochloride |  | 38 | Vismodegib |
| 9 | Topotecan hydrochloride |  | 39 | Imatinib |
| 10 | Regorafenib |  | 40 | Idarubicin hydrochloride |
| 11 | Allopurinol |  | 41 | Mercaptopurine |
| 12 | Arsenic trioxide |  | 42 | Altretamine |
| 13 | Decitabine |  | 43 | Uracil mustard |
| 14 | Cladribine |  | 44 | Tretinoin |
| 15 | Exemestane |  | 45 | Lenalidomide |
| 16 | Mitotane |  | 46 | Pipobroman |
| 17 | Capecitabine |  | 47 | Bortezomib |
| 18 | Olaparib |  | 48 | Idelalisib |
| 19 | Dasatinib |  | 49 | Sorafenib |
| 20 | Vemurafenib |  | 50 | Nilotinib |
| 21 | Fluorouracil |  | 51 | Mechlorethamine hydrochloride |
| 22 | Temozolomide |  | 52 | Floxuridine |
| 23 | Carmustine |  | 53 | Cytarabine hydrochloride |
| 24 | Ifosfamide |  | 54 | Dexrazoxane |
| 25 | Anastrozole |  | 55 | Nelarabine |
| 26 | Clofarabine |  | 56 | Megestrol acetate |
| 27 | Celecoxib |  | 57 | Axitinib |
| 28 | Pemetrexed disodium salt |  | 58 | Raloxifene |
| 29 | Pazopanib hydrochloride |  | 59 | Afatinib |
| 30 | Vandetanib |  | 60 | Ixabepilone |

|  | **Drugs** |  |  | **Drugs** |
| --- | --- | --- | --- | --- |
| 61 | Thiotepa |  | 91 | Bosutinib |
| 62 | Methoxsalen |  | 92 | Ceritinib |
| 63 | Thalidomide |  | 93 | Docetaxel |
| 64 | Pentostatin |  | 94 | Plicamycin |
| 65 | Pomalidomide |  | 95 | Oxaliplatin |
| 66 | Bendamustine hydrochloride |  | 96 | Daunorubicin hydrochloride |
| 67 | Mitoxantrone |  | 97 | Irinotecan hydrochloride |
| 68 | Ibrutinib |  | 98 | Cabazitaxel |
| 69 | Pralatrexate |  | 99 | Bleomycin sulfate |
| 70 | Romidepsin |  | 100 | Zoledronic acid |
| 71 | Aminolevulinic acid hydrochloride |  | 101 | Doxorubicin hydrochloride |
| 72 | Lomustine |  | 102 | Fulvestrant |
| 73 | Procarbazine hydrochloride |  | 103 | Paclitaxel |
| 74 | Gemcitabine hydrochloride |  | 104 | Vinorelbine tartrate |
| 75 | Chlorambucil |  | 105 | Melphalan hydrochloride |
| 76 | Carboplatin |  | 106 | Etoposide |
| 77 | Gefitinib |  | 107 | Trametinib |
| 78 | Methotrexate |  | 108 | Vinblastine sulfate |
| 79 | Enzalutamide |  | 109 | Temsirolimus |
| 80 | Omacetaxine mepesuccinate |  | 110 | Abiraterone |
| 81 | Cabozantinib |  | 111 | Tamoxifen citrate |
| 82 | Estramustine phosphate sodium |  | 112 | Dabrafenib mesylate |
| 83 | Valrubicin |  | 113 | Vincristine sulfate |
| 84 | Everolimus |  | 114 | Imiquimod |
| 85 | Triethylenemelamine |  | 115 | Crizotinib |
| 86 | Ponatinib |  | 116 | Epirubicin hydrochloride |
| 87 | Lapatinib |  | 117 | Teniposide |
| 88 | Carfilzomib |  | 118 | Sirolimus |
| 89 | Dactinomycin |  | 119 | Plerixafor |
| 90 | Amifostine |  |  |  |

**Table S2.** Synergy between HK2 inhibition and DPI.

|  | Combination index (CI)^a^ at | | | |
| --- | --- | --- | --- | --- |
|  | **IC50** | **IC75** | **IC90** | **IC95** |
| **Hep3B** | 0.0167 | 0.0038 | 0.0009 | 0.0003 |
| **Huh7** | 0.0490 | 0.0036 | 0.0028 | 0.0024 |
| **JHH7** | 0.0339 | 0.0197 | 0.0231 | 0.0262 |
| **HepG2** | 0.2752 | 0.1727 | 0.1084 | 0.0789 |
| **JHH5** | 0.0535 | 0.0027 | 0.0009 | 0.0005 |

^a^ Cell growth and viability were measured by the MTT assay. CI was calculated using the CompuSyn software (Combosyn, Inc) based on the Chou-Talalay method. CI < 1, synergism; CI = 1, additive; CI > 1, antagonism.
